# Labelling experiments in red deer provide a general model for early bone growth dynamics in ruminants

**DOI:** 10.1038/s41598-021-93547-4

**Published:** 2021-07-07

**Authors:** Teresa Calderón, Walter Arnold, Gabrielle Stalder, Johanna Painer, Meike Köhler

**Affiliations:** 1 Institut Català de Paleontologia Miquel Crusafont (ICP), Edifici Z, Universitat Autònoma de Barcelona, C/ de Les Columnes, s/n., 08193 Bellaterra, Barcelona Spain; 2 Research Institute of Wildlife Ecology, University of Veterinary Medicine, Vienna, Austria; 3 ICREA, Pg. Lluís Companys 23, 08010 Barcelona, Spain

**Keywords:** Bone, Wide-field fluorescence microscopy, Ecology

## Abstract

Growth rates importantly determine developmental time and are, therefore, a key variable of a species' life history. A widely used method to reconstruct growth rates and to estimate age at death in extant and particularly in fossil vertebrates is the analysis of bone tissue apposition rates. Lines of arrested growth (LAGs) are of special interest here, as they indicate a halt in bone growth. However, although of great importance, the time intervals between, and particularly the reason of growth arrests remains unknown. Therefore, experiments are increasingly called for to calibrate growth rates with tissue types and life history events, and to provide reliable measurements of the time involved in the formation of LAGs. Based on in vivo bone labelling, we calibrated periods of bone tissue apposition, growth arrest, drift and resorption over the period from birth to post-weaning in a large mammal, the red deer. We found that bone growth rates tightly matched the daily weight gain curve, i.e. decreased with age, with two discrete periods of growth rate disruption that coincided with the life history events birth and weaning, that were visually recognisable in bone tissue as either partial LAGs or annuli. Our study identified for the first time in a large mammal a general pattern for juvenile bone growth rates, including periods of growth arrest. The tight correlation between daily weight gain and bone tissue apposition suggests that the red deer bone growth model is valid for ruminants in general where the daily weight gain curve is comparable.

## Introduction

Growth rate is a fundamental variable of an individual’s life history^1–3^ as it plays a major role in the timing of onset and offset as well as in the duration of life history events. In mammals, growth rate is particularly high early in life when the individual is under time constraints to attain a minimum weight and size for reproduction^4^, and it decreases after maturity when the animal ages^5^. The rate at which an individual grows depends on both intrinsic (e.g. genetics, age, disease)^6,7^ and extrinsic factors (e.g. cyclical and non-cyclical changes in resource availability and thermal conditions)^8,9^.

Life history events like birth and weaning fall within the category of non-cyclical episodes that potentially affect growth rate temporally via resource bottlenecks (transitions in the nutritional environment accompanied by modifications in energy metabolism). This is especially the case for early life history events which are characterised by profound changes in source and type of nutrients obtained during the period of maximum growth, i.e. a change from placental nutrient supply (glucose, amino acids and lactate) to maternal milk (high fat–low carbohydrate diet), and subsequently a change from milk to autonomous feeding (in herbivores typically low fat–high carbohydrate)^10^. Later life history events like sexual maturity, reproductive cycles, or onset of senescence involve changes in the allocation of acquired resources to reproduction and maintenance^11^.

A widely used approach to identify and evaluate changes in growth rate is bone histology^12^. One of the methods is the analysis of bone tissue types^13^. Times of decrease in resource availability or increase in energetic requirements are identified by bone growth marks leaving lines of arrested growth (LAGs) when growth stops, annuli (bands of slow growth) when growth is almost residual, or transitions from fast to slower growing tissue when growth rate is more or less reduced. Growth marks are either cyclical reflecting seasonal changes in growth rate, or non-cyclical supposed to be caused by life history events, unusual periods of resource constrains, or illness. Non-cyclical growth marks have been convincingly attributed to birth (neonatal line)^14^, or weaning in skeletochronological studies, where such “extra lines” have been found (“weaning line” by Morris^15^; “discordance” by Castanet et al.^16^; “adhesion line” by Klevezal and Kleinenberg^17^; “dark band” by Barker et al.^18^). However, experimental confirmation that these non-cyclical growth marks are indeed due to the supposed early life history events is a still pending matter, particularly for weaning.

A further issue that needs to be addressed is how much time is compressed within a LAG or an annulus. A method frequently applied in skeletochronology to study this is, for instance, to divide the space between cyclical LAGs by the days of a year, and use this daily growth distance as a measure for estimating the number of days elapsed for forming a particular growth rate^19,20^. However, this approach is false whenever growth marks produced by growth arrest imply periods of complete halt when no change in space is formed over time.

In vivo fluorochrome labelling is currently the most precise method to gain insight into bone growth dynamics, to assess the timing (onset and offset) of growth mark formation (LAGs, annuli, resorption lines), and to reliably calibrate life history events with variations in growth rate and their corresponding growth marks. Nevertheless, labelling is a costly and time-consuming technique and therefore rarely applied. Fluorochrome labelling has previously been used to test the validity of skeletochronological methods in mandibles of small carnivores^21^ and long bones of primates^16^, to understand the relationship between growth rate and bone tissue type in chicks of different bird species^22–25^, to evaluate the pattern of bone remodelling in small mammals^26^, to assess periodicities of regular incremental markings^27–29^ and variation of apposition rates^30^ in dental tissue and for standardisation of procedures and other technical aspects of labelling^31,32^. Bone labelling, however, has never been used to calculate the time at which possible sequels of early life history events form in bone tissue or to estimate the time span enclosed in a growth mark (LAG or annulus).

Here we used in vivo labelling to analyse the impact of life history events on bone tissue formation and to reconstruct early growth rate in a long lived mammal, the red deer. We sampled an ontogenetic series of six red deer calves to obtain a tissue sequence that enables the detection of early events, reflected initially in bone tissue, but eroding at later ages by expansion of the medullary cavity. In this way, we describe how birth and weaning events affect growth in general (i.e. daily weight gain; DWG) and bone growth in particular (tissue apposition, drift and resorption), give and account of the growth marks these events leave in all limb bones, and settle the time of onset and duration of the impact of these early life history events on bone tissue growth rate.

## Results

### Body mass

**Figure 1:**
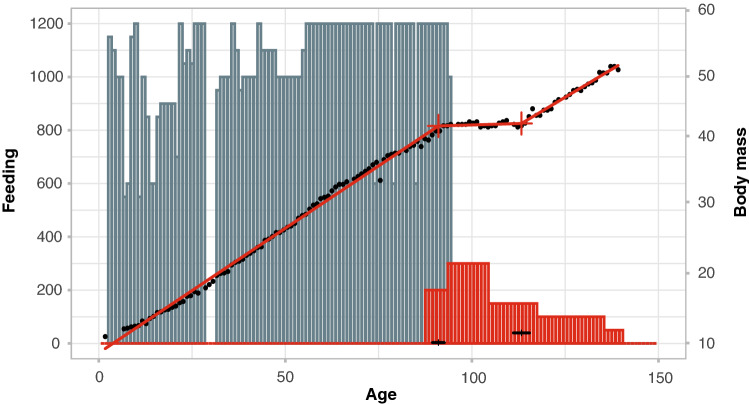
Daily body mass (in kilograms) (black dots) of the hand-raised female ID-4 and daily intake of food (blue and pink bars) plotted against age in days. Crosses indicate breakpoints estimated by segmented regression, horizontal bars above the x-axis 95% confidence intervals of breakpoint locations. Blue bars denote the amount of milk substitute ingested per day (millilitres); red bars the amount of fodder in (milligrams per litre). Slopes of regressions divided by breakpoints differed significantly (F_(2,123_) = 148.69, p < 0.0001; linear modelling without breakpoints yielded a significantly worse fit (model without breakpoints AIC = 556.58; model with breakpoints AIC = 42.36; ∆AICc = 514, p < 0.0001).

Continuous measurements of body mass were available only from one individual (Fig. 1). Starting from a birth mass of 10 kg, this specimen grew linearly with a rate of 0.38 kg per day until weaning at day 91. After weaning, body mass remained constant for 22 days. Growth resumed thereafter, but at a slightly lower rate (0.34 kg per day, difference between slopes of regression before the first and after the second breakpoint (Fig. 1, t = − 2.63, p = 0.01).

### Bone growth rates

To calculate bone growth rates we used the bone appositional area between labels instead of linear measurements (see “Materials and methods”). The complete record of measured areas can be found in Supplementary Table 1, while the record of apposition rates and canal density can be found in Supplementary Table 2.

**Figure 2:**
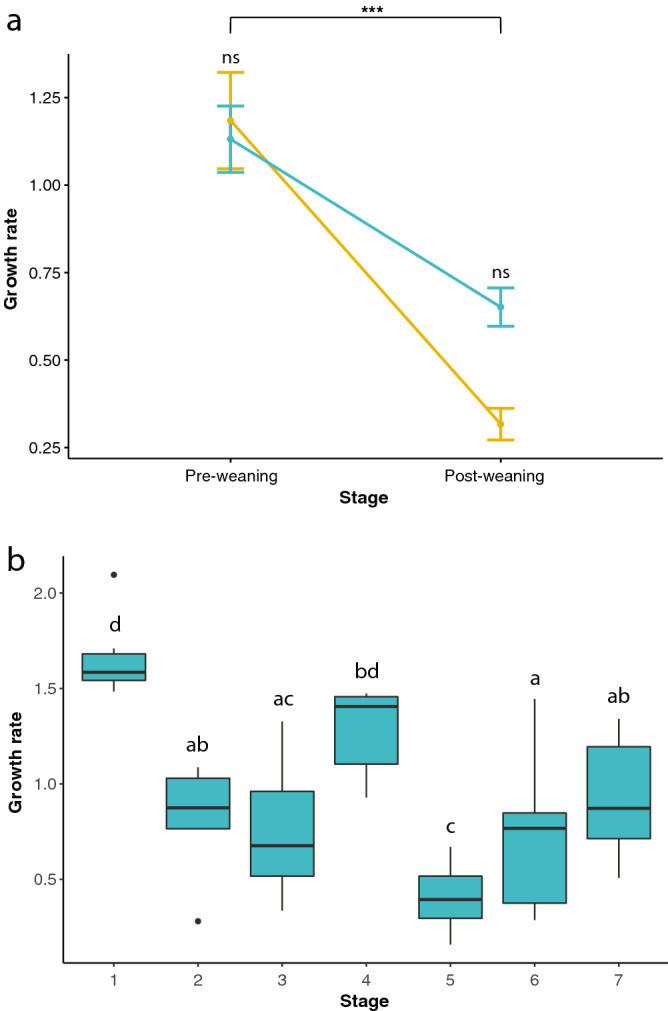
Average growth rates of the six bones analysed (in mm^2^/day) (**a**) for the pre- and post-weaning stages of calves nursed by their mothers (C1; orange colour, means ± standard errors of means) and hand raised calves (C2; blue colour) and (**b**) for the seven growth stages of hand-raised specimens (C2; blue colour). (**b**) Box plots of growth rates of C2 individuals with finer resolution of time (stage 1: birth–3 days; stage 2: 3–15 days, stage 3: 3–30 days; stage 4: 30 days–13 weeks; stage 5: 13–15 weeks; stage 6: 15–17 weeks and stage 7: 17–23 weeks). In graph (**a**), ns: no significant differences. In graph (**b**), different letters above each point indicate significant differences (when groups show different letters) and non-significant differences (when groups share a letter) between groups according to Tukey post-hoc comparisons (p < 0.05).

We compared the growth rate of all six limb bones considering the entire interval from birth to 23 weeks and we did not find significant differences among them (ANOVA; F_(5,60)_: 1.193; p = 0.323). The quantitative analysis revealed the complete record of growth rate of the six limb bones analysed for hand raised (C2) red deer calves and those nursed by their mother (C1) over the first 6 months of life (Fig. 2a). Both groups showed high growth rate in the pre-weaning stage (birth–13 weeks), and substantially lower growth rate in the post-weaning stage (13–23 weeks; two-way ANOVA, effect of stage, F_(1,74)_ = 36.06, p < 0.001; difference between C1 and C2, F_(1,74)_ = 1.54, p = 0.218; difference between slopes, F_(1,74)_ = 2.60, p = 0.111). For the C2 calves, we had more detailed information and could analyse growth rates on a finer time scale over the first 6 months of life. We found significant differences among seven different age stages (stage 1: birth–3 days; stage 2: 3–15 days, stage 3: 3–30 days; stage 4: 30 days–13 weeks; stage 5: 13–15 weeks; stage 6: 15–17 weeks and stage 7: 17–23 weeks) (Fig. 2b; ANOVA, F_(6,59)=_19.11, p < 0.001). Pairwise comparisons demonstrated the degree of differentiation between age stages. Growth rates were highest during stages immediately posterior to birth (1: 1–3 days) and previous to weaning (4: 30 days–13 weeks), and lowest immediately posterior to weaning (5: 13–15 weeks). This minimum growth rate corresponded to the almost null apposition of bone (see below) and was characterized by the presence of a growth mark. Growth rates recovered again thereafter (6: 15–17 weeks, 7: 17–23 weeks).

### Cumulative bone tissue apposition

**Figure 3:**
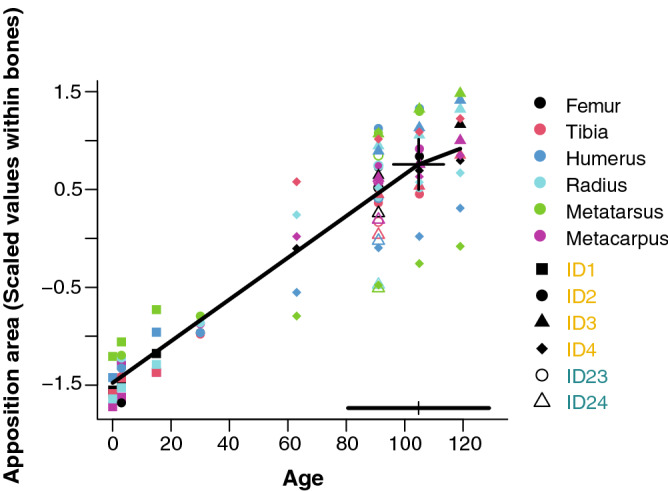
Cumulative apposition areas of bone tissue in six different limb bones during early life (scaled values of bone apposition area in mm^2^, plotted against age in days), of calves nursed by their mothers (C1: ID-23, ID-24), and hand-raised calves (C2: ID-1, ID-2, ID-3, ID-4). The cross indicates a breakpoint estimated by segmented regression, the horizontal bar above the x-axis indicates the 95% confidence interval of breakpoint location in which the weaning event (occurred at the age of 90 days) is included. Slopes of regression below and above the breakpoint differed significantly (F_(1,57_) = 6.25, p = 0.015; linear mixed modelling without a breakpoint yielded a significantly worse fit (model without breakpoints AIC = 32.14; model with breakpoints AIC = 11.22; ∆AICc = 19.92, p < 0.0001).

Data from cumulative bone apposition areas measured between successive labels in six different bones revealed fast growth up to an age close to weaning (between 90 and 110 days according to the confidence interval) and a slightly slower growth thereafter (Fig. 3). Unlike the hand-raised calves (C2), labelled several times, calves nursed by their mother (C1) were labelled only at the age of 91 days. However, at this age bone apposition did not differ significantly between both groups (F_(1,3)_ = 2.68, p = 0.200).

**Figure 4:**
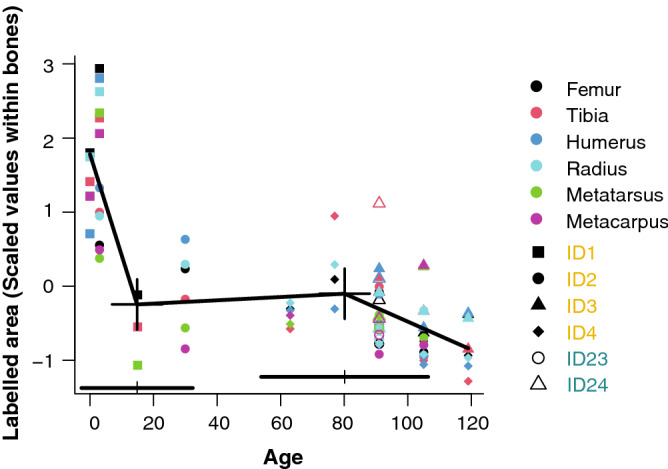
Amount of bone area stained with fluorochromes for each of the labels in six different limb bones. Plotted are scaled values of deposition areas in mm^2^ of calves nursed by their mothers (C1: ID-23, ID-24), and hand-raised calves (C2: ID-1, ID-2, ID-3, ID-4. Crosses indicate breakpoints estimated by segmented regression, horizontal bars above the x-axis 95% confidence intervals of breakpoint locations. Slopes of regressions divided by breakpoints differed significantly (F_(2,46_) = 14.33, p < 0.0001; linear modelling without breakpoints yielded a significantly worse fit (model without breakpoints AIC = 179.66; model with breakpoints AIC = 132.59; ∆AICc = 47.07, p < 0.0001).

Further, we quantified the deposited area of each fluorochrome injection. This approach provided a quite narrow and, hence, temporally discrete bone appositional rate. It corroborated the result found with the cumulative bone apposition areas, but delivered a more detailed picture (Fig. 4). The area of pigment deposition decreased substantially after birth, resumed until weaning and was followed by a second, less pronounced decrease.

**Figure 5:**
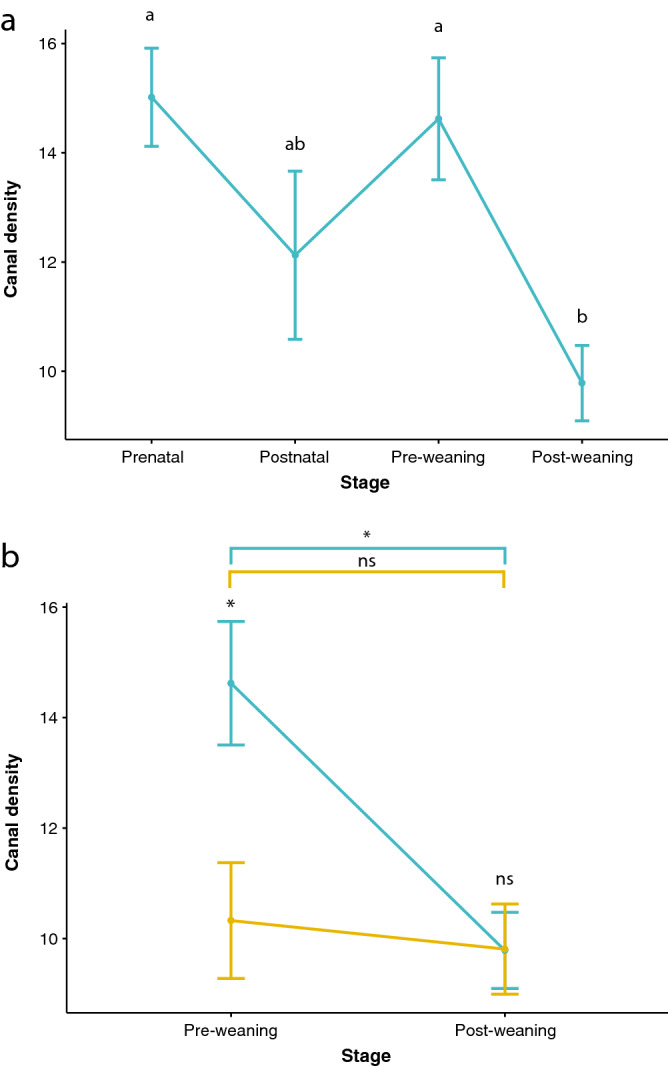
Representation of canal density (mean ± SE in % of the total area contained in a square of 0.370 mm^2^) (**a**) by ontogenetic periods of hand-raised calves (C2): prenatal (before birth), post-natal (from birth to 30 days), pre-weaning (from 30 days to 13 weeks) and post-weaning (from 13 to 23 weeks) and (**b**) for the pre-weaning (from 30 days to 13 weeks) and post-weaning (from 13 to 23 weeks) periods of both groups of calves (C1 and C2). Specimens staying with their mothers (C1) are represented in orange; hand-raised specimens (C2) are represented in blue. In graph (**a**), different letters above each point indicate significant differences (when groups show different letters) and non-significant differences (when groups share a letter) between groups according to Tukey post-hoc comparisons (p < 0.05). In graph (**b**), ns: no significant differences.

Lastly, we calculated the canal density, another independent indicative of bone deposition rate^12^) for four ontogenetic periods: just before and after birth, and just before and after the weaning (Fig. 5). Results revealed differences between these ontogenetic periods in hand-raised specimens (Fig. 5a; ANOVA, F_(3,41)_ = 5.283, p = 0.004). When comparing data from the two groups of calves, we found differences also conditioned by feeding regimes (Fig. 5b; two-way ANOVA, F_(1,50)_ = 4.503, p = 0.038). Both groups (C1 and C2) experiment a decreasing trend between pre- and post-weaning period, but in a different way: the group of hand-raised calves (C2) show a higher density than calves nursed by their mothers (C1) in the pre-weaning period while both groups show almost the same density after the weaning event (Fig. 5b).

### Histological representation of growth rate variation

All the detailed histological descriptions of every bone section can be found in Supplementary Material.

**Figure 6:**
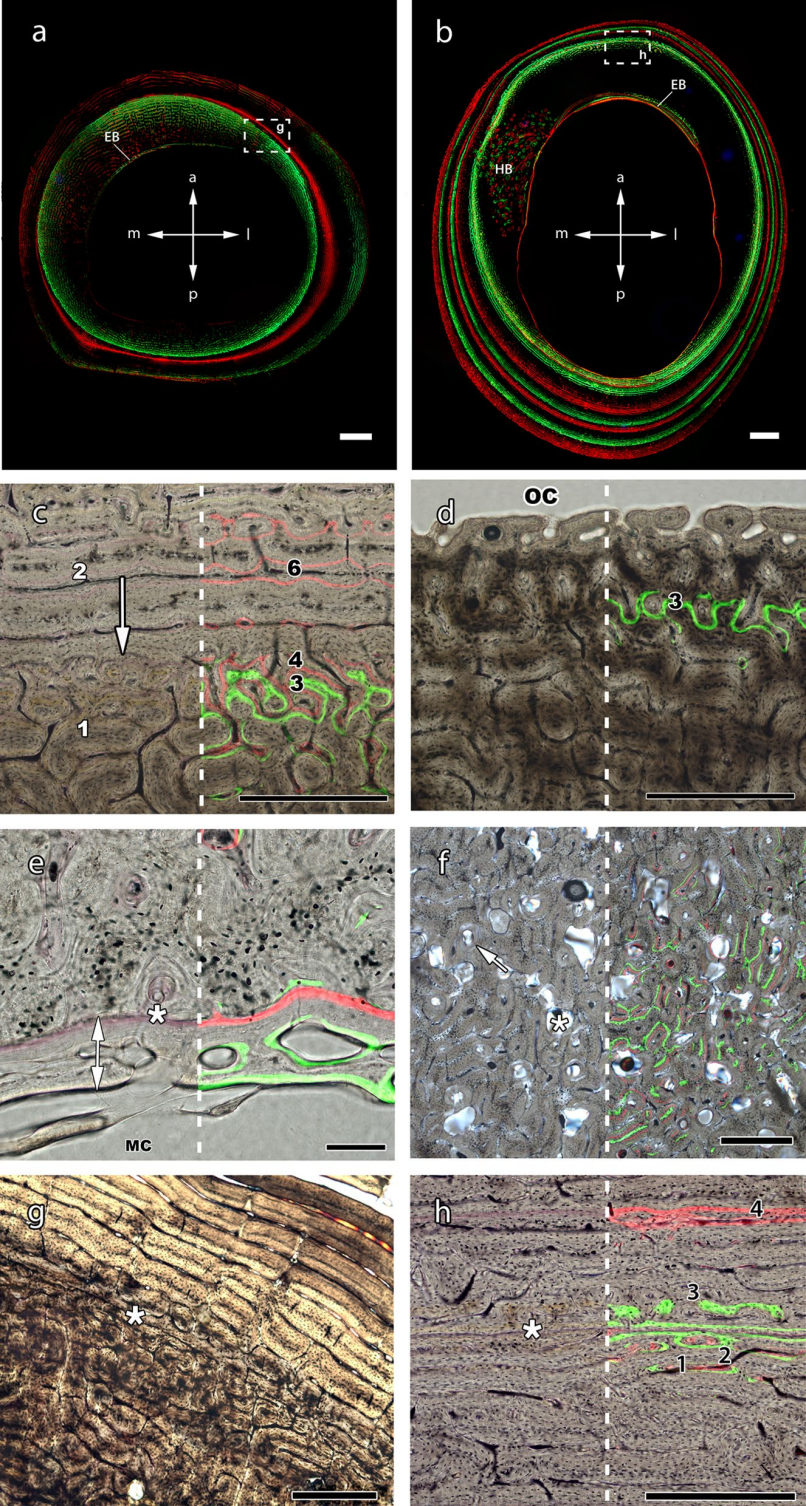
Limb bones under fluorescent light (**a**,**b**) and their histological details under transmitted (**g**) and combined transmitted and fluorescent light (**c**–**h**). (**a**) Bone expansion (bone drift) towards the postero-lateral side of the tibia of a 15-weeks old individual (ID-2). An initial cortical layer (endosteal bone: EB) formed around the antero-medial part of the medullary cavity as a consequence of bone drift. In this sector, later tissue (red label 30 day) has locally been arrested (upper part of first red label) to remodel the shape of the bone. Inner green label: 3rd day after birth; red label: 30 days after birth; outer green label: 13 weeks after birth; outer red label: 15 weeks after birth. Scale 2 mm. (**b**) Bone expansion towards the posterior side of the humerus of a 43 weeks old individual (ID-3). The EB appears in the opposite site while a section of Haversian bone (HB) can be seen towards the medial sector. Inner green label: 13 weeks after birth; red label: 15 weeks after birth; second green label: 17 weeks; second red label: 23 weeks; subsequent labels were not considered in this study. Scale 2 mm. (**c**) Abrupt shift of the FLC from reticular pre-weaning tissue (white 1) to a plexiform-laminar post-weaning tissue (white 2) in the anterior region of the metacarpus of a hand-raised individual (ID-3). A resorption process has eroded the deposition between labels 4 and 6. The white arrow indicates the transition between pre- and post-weaning periods. 3: label deposited at 13 weeks; 4: label deposited at 15 weeks; 6: label deposited at 23 weeks. Scale 0.5 mm. (**d**) Uninterrupted deposition of tissue in the anterior part of the metatarsus of an individual nursed by its mother (ID-24). 3: label deposited at 13 weeks. Scale 0.5 mm. (**e**) Endosteal bone (white arrow) in the anterior sector of the metatarsus of ID-1 delineated by a cement line (asterisk). Red label: alizarin red deposted at the age of 3 days; Green label: calcein green deposited at the age of 15 days. Scale 0.1 mm. (**f**) Detail of a forming Haversian bone in the posterior sector of the metatarsus of ID-3. Resorption cavities are marked with an asterisk and cement lines of secondary canals with an arrow. (**g**) Birth LAG (white asterisk) in the antero-lateral part of the tibia of ID-2. A more disorganized tissue before birth (down) and a more organized tissue after birth (up) indicate a slowdown in the deposition rate. Scale: 0.5 mm. (**h**) Weaning LAG (white asterisk) in the anterior part of the humerus of ID-3. Black numbers indicate the different labels: 1: green label deposited at 13 weeks; 2: red label deposited at 15 weeks; 3: green label deposited at 17 weeks; 4: red label deposited at 23 weeks. The absence of tissue deposited corresponds to the period between 13 and 17 weeks, making barely visible the 15-week label (number 2). Scale 0.5 mm.

Every bone of the individuals here analysed was still actively growing at the time of death. The patterns of bone apposition displayed by the same label over different bone sections revealed different rates of cortical growth. Thus, a wide and spaced arrangement of successive labels indicated areas with elevated rates of bone apposition (fast-growing areas), while a compact arrangement of successive labels revealed areas of low rates of bone apposition (slow-growing areas) (Fig. 6a,b). Regardless of area, C2 individuals showed a general period of low apposition that starts at 13 weeks and lasts until 17 of age.

#### Bone tissue formation

Primary bone mainly consisted of woven-parallel complex (see “Materials and methods” for nomenclature), specifically of fibro-lamellar tissue (FLC). Prenatal tissue showed a greater amount of primary parallel-fibered bone than postnatal tissue (Table 1; Fig. SM1a), which facilitated the visual recognition of this boundary under polarized light in all bones of the individuals here studied. As postnatal bone increased in proportion over ontogeny, the prenatal bone is being resorbed, though even the oldest individuals showed remnants of prenatal tissue (i.e. 43 weeks-old) (Table 1). We distinguished three main types FLC: laminar, plexiform and reticular. Radial orientation predominated in some areas of the tibiae and some longitudinal areas can be recognized in metapodials. These FLC types varied widely throughout the bone matrix (Table 1). Bones of C2 individuals experienced a shift in the type of FLC tissue towards a more organized pattern after weaning (i.e. 13 weeks of life) (Table 1; Fig. 6c) while bones of C1 individuals show a uniform deposition (Fig. 6d). Moreover, specimens of both groups showed local differences in transversal area (i.e. fast-growing areas frequently showed radial and plexiform FLC, while laminar and plexiform FLC was common in areas of slow-growth)*.*

**Table 1:** Description of thin-sections.

**Perinatal growth**
Rounded shape and narrow bone cortex
The medullary cavity occupies a high percentage of the cross-section
Cortex formed mostly by prenatal tissue with reticular arrangment (FLC with high percentage of PFB in the prenatal area)
Absence of annulus and LAGs
Endosteal bone present in radius and metapodials at the age of three days in the anterior part (metapodials) and in the medial and lateral areas (radius)
Haversian bone absent
**Pre-weaning growth**
Bone cortex slightly irregular but still rounded
Medullary cavity area increased
Prenatal bone still present. Variable types of FLC in group C2. Mostly plexiform in group C1
Growth marks bordering the prenatal area in radii of C1 and tibiae, radii and metatarsii of C2
Endosteal bone starts to form at 13w in proximal bones and tibia
Incipient Haversian bone in the *linea aspera* (femur), medial part (tibia and radius) and posterior area (metapodials)
**Post-weaning growth**
Shape similar to adults. Thick cortex
Large medullary cavity except for radius and metapodials
Low portion of prenatal tissue. Shift in FLC arrangement in group C2. Mostly plexiform tissue in group C1
Growth marks bordering the prenatal area in both groups (C1 and C2) and between the 13th and the 17th weeks label only in the C2 group. Growth marks with cortical resorption bordering the pre-weaning area in tibia and radius
Well-developed endosteal bone but already reduced by resorption to few small areas
Well-developed Haversian bone in the *linea aspera* (femur) and some prenatal areas (rest of the bones), which continues spreading around the shaft

*FLC* fibrolamellar complex, *PFB* parallel fibered bone, *LAG* line of arrested growth, *C1* calves left with their mothers, *C2* hand-raised calves.

Secondary bone such as endosteal tissue and Haversian systems was present in the limb bones from an early age onwards (Table 1). Although growing centripetally, the endosteal tissue found in our sample was deposited following the same pattern as primary tissue (i.e. FLC). A woven-bone scaffold started to form and, subsequently, it was refilled with lamellar tissue following different FLC arrangements (Fig. 6e; Fig. SM2f, SM3f). Deposition of Haversian bone started at the age of 15 weeks; their presence was restricted to particular areas depending on the bone (Fig. 6f).

#### Indicators of growth arrest

There was a correlation of growth marks with birth and weaning events. Describing incomplete circumferences, these growth marks did not follow the entire former bone circumference, but they only formed within the areas of low apposition.

A partial LAG or an annulus (depending on the bone and the location, but usually in the slow-growing areas) appeared in the moment of birth bordering the prenatal area (Fig. 6g). This growth disruption was associated with a local decrease in growth rate (Fig. 4) until approximately 3-days after birth. We found another growth disruption after the moment of weaning (13 weeks) in the slow-growing areas of all bones of hand-raised individuals (C2) older than 15 weeks (Figs. 3, 4). This disruption was represented by a partial LAG located between the labels deposited at 15 and 17 weeks (Fig. 6h) or shortly after the 15-weeks label in all the bones of C2 individuals but only in a the radius of a C1 individual (Fig. SM3d). Taking this into account we estimated that limb bones of C2 group arrested growth for 1–2 weeks within the slow-growing areas in response to weaning. There was no evidence of LAGs in the fast-growing areas of the shaft, though in most of the specimens there was no increase in bone area from 13 to 15 weeks.

These birth and weaning growth marks were accompanied by local cortical resorption in the slow-growing areas of radii, tibiae and metapodials of C2 individuals and of the radius of one C1 individual (Fig. SM3d). A scalloped interruption of the tissue in combination with a growth mark was visible in these areas. In addition to these intervals, and apparently independent of any life history event, there were some marks on the lateral side of the radius both in C1 and C2 individuals.

## Discussion

Previous in-vivo fluorochrome labelling experiments were aimed at testing the reliability of skeletochronology, generally in reptiles and mammals^16,21,33,34^. However, a few studies focused on quantifying bone tissue growth rates using juvenile (and, hence, rapidly growing) birds. Despite the generally fast growth rates observed, these studies found both quantitatively and qualitatively much variation in absolute growth rates in different bones and even within each tissue type^22–25^; nevertheless, they have so far failed to provide a uniform and general picture of the dynamics of bone growth during early ontogeny in vertebrates. This is essentially due to the design of the studies. As they were not aimed at determining the timing and duration of the observed variations nor their correlation with early life history events (hatching in birds, birth and weaning in mammals), but rather at finding a correlation between visually classifiable bone tissue types and quantifiable tissue growth rates (testing Amprino’s rule). Here, by fine-scheduled, high-resolution labelling, we describe a general pattern for juvenile bone growth rates in red deer as an almost continuous decrease with age. This trend shows two events of growth disruption that coincide with the life history events birth and weaning. In this way, results on bone tissue apposition and body mass gain, suggest that the bone development from birth to post-weaning in deer calves represents indeed a general 4-stages pattern for ruminants, described by birth (three days of growth decrease/arrest), the period between birth and weaning (vigorous growth), weaning (growth decrease/arrest for 1-to-2 weeks), and post-weaning (modest growth resumption).

A birth line has been first identified and described by Nacarino-Meneses and Köhler^14^ as neonatal line, homologous to the birth line found in teeth^35^, by superimposition of bone sections of aged individuals in several *Equus* species, evidencing the association between this non-cyclical growth mark and the event of birth in mammals. Our labelling results corroborate that the first growth mark (either a LAG indicating growth arrest or an annulus indicating a change in growth rate) deposited in the limb bones of red deer correspond to the birth event. The fact that we found both LAGs and annuli forming a neonatal line, as Nacarino-Meneses and Köhler^14^ did in *Equus*, indicates that not all long bones of a same individual uniformly arrest growth (LAGs); some do not stop but only decrease growth rate to a minimum (annuli). This corroborates previous findings that tissue growth rates vary both within and among long bones, which is especially obvious between legs and wings of birds^22–25^.

The boundary between pre- and postnatal tissue in red deer calves is further emphasised by an abrupt transition in tissue type from predominantly parallel-fibered prenatal bone to postnatal fibro-lamellar cortex with lower proportions of parallel-fibered bone, indicating acceleration in growth rate after the event of birth. This pattern is comparable to that found in equid foals^14^ and elephantidae^36^ and opposed to the pattern found in dinosaurs and reptiles^37,38^.

A weaning line was found in the jawbones of hedgehogs^15^. The dark band found by the authors was attributed to the reduced growth caused by the food disruption of weaning. Posteriorly, Castanet et al.^16^ describe a discontinuity in the inner part of the long bones that may indicate the weaning event due to the date of deposition and which they did not consider for age estimation. In a similar way, the “dark line” discounted in skeletochronology of yellow-pine chipmunks by Barker et al.^18^ could correspond to a weaning line; alternatively, their “extra adhesion line” might instead represent a neonatal line. However, Barker et al.^18^ provided no description so that an exact determination is impossible.

Results provided by our labelling schedule evidences that this early growth mark, mainly present in the hand-raised individuals (C2 group), corresponds to the weaning process. In addition to the growth mark, the transition from pre- to post-weaning period is marked by a shift in the type of FLC that reflects the change from high to low bone growth rates^25^ in hand-raised specimens (C2). The weaning process is also the responsible of the temporal stagnation of the daily body mass gain during approximately 4 weeks (Fig. 1).

Unlike the hand-raised specimens (C2), we could not weigh the C1 group daily because these individuals were untamed. Thus, we had to restrict the weight measurements to the labelling at approximately weaning and at death. Notwithstanding, the fact that we found a weaning LAG in most of the specimens of the C2 group and only in a single element of C1, could be due to either the different pre-weaning feeding regimes or to the less gradual and more abrupt artificial weaning experienced by the hand-raised group. As in all ruminant artiodactyls, the digestive system of red deer calves changes during early life, specifically during the pre-weaning period. The rumen development is highly conditioned by the addition of vegetal material during the pre-weaning period. When the rumen is fully developed and functional (i.e. around 12 weeks), calves are able to feed entirely on vegetal materials^39^. Despite the gradual modification of diet before weaning in the hand-raised deer (i.e. a decreasing amount of milk and an increasing amount of vegetal material), it is possible that this shift could have been not smooth enough as in naturally weaned calves. As a consequence, the rumen could have underwent an incomplete development, making difficult the absorbance of the only-vegetal feeding and hence, causing a marked growth disruption.

The negative effects of weaning on growth rate of juveniles is widely studied in domestic breeds^40^. This is because domestic species experience an abrupt weaning that often starts too early compared to the gradual weaning of wild animals (Holland et al.^41^ on foals; Enríquez et al.^42^ on beef calves). Therefore, the improvement of the weaning process with the aim of maximizing production efficiency^43^ is in the foreground of many studies on farm animals. In this way, farm managers use to employ artificial feeding (i.e. milk-based diet), as we have employed in the hand-raised calves (C2), as a method for improving the production efficiency. Various studies^44,45^ have demonstrated that milk replacer allows for an accelerated growth rate in piglets. This supports the different pre-weaning vascular density found between our hand-raised calves (C2) and those fed by their mothers (C1). In our case, this is conditioned by the composition of powdered milk, richer in nutrients than natural deer milk^46, 47^. This high energy diet fed to hand-raised animals most likely led to a fast periosteal bone apposition generating a highly vascularized matrix (Fig. 5b)^12,48^.

However, the accelerated growth triggered by the high nutrient diet of powdered milk could have caused long-term effects. Fast calf growth during lactation positively determines adult body size and mating success^49,50^. Moreover, larger calves are more able to tolerate nutritional deficiencies and harsh winter conditions^51^, even in farmed environments^52^. In contrast, accelerated growth could also cause negative effects. Some authors^53,54^ have explored the mismatch between a low prenatal and high postnatal growth conditioned by milk substitutes in different groups of mammals (mice and cattle, respectively). Based on life history theory, they concluded that fast growth could potentially lead to reduction in longevity, and a decrease in fertility and metabolic activity. For these reasons, it is important to have a good understanding of this pre-natal stage as the accelerated growth of C2 individuals feeding on milk replacer could cause similar detrimental long-term responses.

Our labelling study provides, for the first time an estimate of the duration of non-cyclical LAG formation during weaning: decrease or even cessation of bone growth during this event lasts 1 or 2 weeks depending on the nutritional conditions (environmental food availability and conversion of acquired nutrients into energy), which can lead either to a rather smooth decrease in bone growth rate in naturally nursed, healthy calves or to an abrupt deposition of a non-cyclical partial LAG under conditions of artificial weaning. The weaning LAG is especially visible where the direction of radial bone growth is constrained by shape (biomechanics) through growth arrest, and is almost imperceptible where bone growth is only reduced but not arrested^55^. Accordingly, the expression (imperceptible, incomplete or complete) of LAGs and annuli within long bones is conditioned by bone growth rates: the slower the rate the easier the formation of rest lines. The absence of cyclical marks in juvenile dinosaurs led to similar conclusions^56,57^.

Additionally to the growth marks caused by birth and weaning events, we relate structures formed by cortical resorption, generally termed “dark lines” and attributed to temporary changes of bone deposition^58^, to growth arrest because of the frequent incidence of these resorption lines with non-cyclical rest lines. Accordingly, we consider these darks lines as LAGs. The fact that resorption in bones is restricted to marked drifts indicates that this phenomenon is related to changes in shape. LAGs deposited at the age of birth and weaning and associated to resorption result from reduced bone apposition triggered by these events combined with a concomitant biomechanical re-adjustment of shape. In some bones, especially in the radius, such biomechanical adjustments are not restricted to these events but happen throughout ontogeny, leading to deposition of several non-cyclical partial LAGs where the bone faces an adjacent bone (here ulna), which limits expansion in this area.

In addition to the histological changes associated with specific life history events, other variations in bone microstructure were observed. Though a bone tissue type can have different absolute growth rates, the factor “tissue type” has been found to have the strongest correlation with bone growth rate^25^. Not surprisingly, hence, postnatal deposited fibro-lamellar bone in our red deer calves varies importantly among skeletal elements. The slowest-growing tissue (longitudinal microarchitecture) is typically found in metapodials, while the fastest-growing tissue is found in tibiae where it frequently shows the unusual and rarely observed radial orientation, which has significantly higher growth rates than other fibro-lamellar bone types^25,59^. This agrees with the dissimilar growth pattern of limb bone length in which the distal segments grow slower after birth than the proximal segments^23^. Moreover, all bones showed internal variation, revealing different local apposition rates and indicating cortical drift toward a specific area depending on the bone, as corroborated the arrangement of the fluorescent labels. Bone drift activity can be also appreciated by the resorption processes occurred in the inner region of the bone (i.e. expansion of the medullary cavity) and the subsequent endosteal deposits of bone found from an early age in our sample. Despite the endosteal bone has been commonly described as a lamellar or parallel-fibered bone tissue^24,60–63^, this work documents an endosteal bone mainly composed by FLC, suggesting fast rates of osteogenesis. Recently, Montoya-Sanhueza et al.^26^ have described in detail the patterns of bone remodelling in long bones of naked mole rat by using in vivo fluorescent labelling. However, the results obtained form that work and for another study including juvenile specimens^64^ showed that the endosteal bone of this species was composed by lamellar tissue. This may due to the fact that these studies are focused in small-size mammals. Further research on the bone microstructure of large mammals is needed to shed light on this issue. Moreover, according to our findings, this structure formed early in ontogeny is partially resorbed short after, highlighting the importance of research focused on the early stages of ontogeny in order to obtain the most complete record of information.

In summary, our labelling study provides, for the first time, a general pattern of juvenile bone growth rates in red deer calves, shows how weaning and different feeding regimes affect bone tissue formation and yields an estimate of the timing of non-cyclical LAG deposition. In this way, the results gathered here allow reconstructing ontogenetic bone growth patterns, provides a firm ground for inferences of growth rates for histological bone structures of hitherto unknown time intervals, and bridges the gap between bone histology and interpretations of life history strategies.

## Material and methods

### Individuals and feeding regime

Six red deer (*Cervus elaphus hippelaphus*) (five males and one female) were used for this study. All animals belonged to the herd of red deer kept at the Research Institute of Wildlife Ecology, University of Veterinary Medicine, Vienna. The deer enclosure contained about 39 ha deciduous oak-beech forest and a meadow of about 6 ha, i.e. provided close to natural living conditions. The animals could forage on natural vegetation in the enclosure and received supplementary pellets and hay. Calves analysed were born between late spring-early summer of 2014, 2015 and 2016. All of them were weighed at birth to the closest 50 g.

Newborn calves were assigned to two groups with different raising conditions and feeding regimes. One group of calves (C1) consisted of two males (ID-23, ID-24) that naturally suckled at their mothers. Both remained in the herd until euthanasia at ages 64 and 24 weeks, respectively. The second group of calves (C2) consisted of two males (ID-1, ID-2), sacrificed at ages 2 and 15 weeks, respectively, and one male (ID-3) and a female (ID-4), both sacrificed when 43 weeks old. Group C2 was initially also left with their mothers to allow for sufficient intake of colostrum, but separated from them within 12 h after birth and hand-raised by experienced animal keepers. Until an age of ~ 4 months C2 animals were individually housed at night, but kept together under supervision for 8 h during daylight. From an age of ~ 4 months onwards, C2 animals were kept together in a ~ 0.75 ha enclosure, separating from the rest of the herd by a fence.

Hand-raised animals were bottle-fed with milk substitute (Alpmil Lämmermilch 22.5% crude protein, 22% crude fat, Garant, Austria). From week 1 to 10 after birth they received an increasing daily ration of milk substitute from ~ 1000 ml (week 1) to 1200–1500 ml (week 10), distributed over 4–5 (week 1), 3 (weeks 3–5), and 2 (weeks 6–10) feeding bouts per day (for individual intakes see Supplementary Table 3). From week 10 onwards the daily amount of milk substitute fed was reduced to introduce the weaning period, and to encourage the animals to feed on pellets (up to an age of 5 months: Lämmerkorn, Garant, Austria; older than 5 months: Trophy Ergänzungsfuttermittel Reh/Rotwild, Garant, Austria) and hay, provided ad libitum. Bottle-feeding was terminated during week 13 after birth (weaning), except for ID-1 that was sacrificed earlier. C2 animals were weighed daily until weaning (13 weeks) and ID-4 until 20 weeks.

### Fluorescent markers

Calves were labelled subcutaneously between birth and age 23 weeks with fluorescent markers (Alizarin complexone and Calcein green), known to get incorporated into growing bone tissue^33^, individuals of group C2 repeatedly (Table 2). Marker solutions were prepared under sterile conditions at the pharmacy of the Vetmeduni Vienna and buffered to 7.4 pH with NaHCO_3_.

**Table 2:** Schedule of alternated calcein green (bold) and alizarin complexone labelling (italic) and the amounts of fluorochrome marker administered (Alizarin complexone 30 mg/kg; calcein green 8 mg/kg).

Age	Marker (ml)					
	Group C2				Group C1	
	ID-1 IPS-88714	ID-2 IPS-88715	ID-3 IPS-93664	ID-4 IPS-88713	ID-23 IPS-109291	ID-24 IPS-109290
Birth	**3.1**					
3 days	*13.3*	**3**				
15 days	**4.8**					
30 days		*19.8*				
9 weeks				**9.1**		
11 weeks				*38*		
13 weeks		**10.3**	**10.9**	**11.3**	**10.9**	**11.2**
15 weeks		*42*	*44*	*43*		
17 weeks			**13.9**	**12.3**		
23 weeks			*70*	*58.5*		

Until the age of about 3 months, all procedures were carried out without anaesthesia, as the hand-reared animals were hand-tame and still small enough to be restrained briefly for injection. Thereafter, all manipulations were performed under deep sedation using 0.1 mg/kg medetomidine (Medetomidine-HCL 2%, magistral formulation, Richter Pharma AG, Vienna, Austria). During sedations, vital parameters of animals were monitored. Sedation was reversed using atipamezole (Narco Stop 5 mg/ml, Richter Pharma AG, Wels, Austria). For euthanasia, animals were deeply anaesthetized with a combination of ketamine (Ketamidor 100 mg/ml, Richter Pharma AG, Vienna, Austria) and medetomidine (2 mg/kg and 0.8 mg/kg, respectively). Thereafter, they received an overdose of a combination of embutramide, mebezonium and tetracaine (T 61, lntervet, Vienna Austria).

Our research questions could only be investigated in a living wild ruminant kept under (semi-) natural conditions. Animals were managed by keepers with decades of red deer experience and were checked daily. Veterinary care was constantly available. All experiments and procedures were approved by the Ethics Committee of the University of Veterinary Medicine, Vienna and the Austrian Federal Ministry of Education, Science and Research in accordance with the Animal Experiments Act 2012 (TVG 2012) (permit numbers (GZ) BMWFW-68.205/0100-WF/II/3b/2014 and BMWF-68.205/0170-WF/V/3b/2016). These experiments were carried out in compliance with ARRIVE guidelines.

### Bone samples

After euthanasia, we prepared from each individual six postcranial bones from sections of the hind- and forelimb (femur, tibia, metatarsus, humerus, radius and metacarpus). The total of 36 bones was sent to the ICP-Institut Català de Paleontologia Miquel Crusafont, Barcelona (Spain) for histologic analysis.

All bones were photographed and measured following standard ICP procedure before sectioning^65^. Bones were sectioned at the central part of the diaphysis. A chunk of approximately 3 cm from the middle of the diaphysis was extracted from each bone (from 1.5 cm above to 1.5 cm below the mid-shaft), degreased and dehydrated by alcohol immersion. Afterwards the chunk was embedded in Araldite 2020 epoxy resin. The block was cut into two halves with a low speed diamond saw (IsoMet low speed saw, Buehler). The cut surfaces were polished with a MetaServ polishing machine and fixed to a frosted glass using epoxy resin. Once the sample was fixed, it was cut with a diamond saw (Petrothin, Buehler) up to a thickness of 100–120 μm and finely polished again to perfect the slide.

### Histological study and label examination

Thin sections were examined under Zeiss Scope A1 microscope with an integrated camera. Firstly, the sample was studied under transmitted and polarized light in order to obtain bone tissue information (e.g. tissue type, vascularity, growth marks). The description of bone tissue types was done following the nomenclature proposed by Prondvai et al.^66^. The FLC was described as longitudinal, laminar, plexiform, reticular or radial regarding according to Francillon-Viellot et al.^67^. Subsequently, the sample was exposed to fluorescent light, alizarin complexone (red) and calcein green with the purpose of obtaining information about the deposited bone at each date scheduled. Images of different sections were taken using Zeiss software and channels were merged in order to obtain a combination of red and green fluorescent image first, and polarized and fluorescent images together. Afterwards, combined section images were aligned and fused using Photoshop CS4 with the purpose of creating the entire surface of the cross-section (see Supplementary material). Although most individuals were labelled several times over a complete year, we focused on the period from birth to 23 weeks with the aim to avoid the overlap with seasonal disruptions (since births took place in June the beginning of winter occurred short time after the 23th week of life).

The complete cross-sectional areas between labels were measured on combined polarized and fluorescent images. For this purpose, the entire area contained in each one of the labels (i.e. fluorescent circumferences along the cortex, Fig. 6c,d), including the medullary cavity, was selected. We included the area of the medullary cavity in each measurement in order not to bias the data as the variation of its size over time cannot be estimated. Also because our purpose is to study variations in deposited area between labels and not the precise amount of area. We measured areas instead of linear measurements (i.e. thickness between labels) as hitherto used^22–25,68^, because area is related to body mass and, hence, better reflects the ontogenetic stages, which was the main purpose of our study. Besides, this method also allowed avoiding the bias caused by asymmetry of bone shafts.

For calculating daily growth rates, the area between labels was divided by the days between injections, which resulted in 7 stages: stage 1: birth–3 days; stage 2: 3–15 days, stage 3: 3–30 days; stage 4: 30 days–13 weeks; stage 5: 13–15 weeks; stage 6: 15–17 weeks and stage 7: 17–23 weeks. Due to the short intervals between the first injections in the specimen ID-4 (Table 2), the fluorochrome lines overlapped in the bone tissue. Hence, it was no possible to calculate growth rates for ID-4 at ages from 9 to 15 weeks. As calves left with their mothers (C1) were labelled only at the age of 13 weeks, we could not measure the growth rates during discrete periods in their ontogeny. However, we could measure the growth rates of the 6 months old specimen ID-24 from birth until the age of 13 weeks and from there to the age of 24 weeks at euthanasia by using the birthmark and the outer circumference of the bone as natural labels.

We quantified the area of the fluorochrome deposited for each label. With that purpose, we created the entire composition of the shaft of red and green fluorescent images separately using Photoshop CS4. Basing on the disposition of the last fluorochrome label deposited (animals were sacrificed 2 days later), we estimated the time of complete pigment deposition and, hence, the time represented by a label as ≈ 48 h in deer. We measured the area of each isolated label in single images using FIJI software. As the increasing diameter of the bone tends to bias these measurements, we standardized the data considering the ratio between the areas of each label (see Supplementary Table 4). In addition to the labelled tissue that was deposited increasing the periosteal area of the shaft at the time of the injection, it was also possible to find concentric stained lamellae in the inner regions of the cortex (i.e. the area that was filling the woven matrix of FLC at that time).

The density of the canals was quantified in the anterior region of all bones for each labelling interval and also for the prenatal period where possible. We used ImageJ software^69^ to obtain the area occupied by vascular canals contained in a limited area of 0.370 mm^2^ using a 20 × lens. Finally, the area occupied by vascular canals was expressed as a percentage in order to facilitate comparisons with other measured areas.

### Statistical analysis

Statistical analyses and charts were performed using RStudio^70^ software. Shapiro–Wilk test and Levene’s test were performed to evaluate the normality and homoscedasticity, respectively, of our data. Once these conditions were confirmed (Saphiro Wilk W > 0.95; Levene test p > 0.05), ANOVA tests were performed to analyse the variation of growth rates over ontogenetic stages and over bones. The variation in growth rates and vascular density between the two groups of calves in different age periods was analysed by applying two-way ANOVA tests. Tukey multiple comparison test was used to establish statistically significant differences between groups. The increase of body mass, bone area and deposition area over time were analysed with segmented regression analysis^71^. To account for different apposition rates in different bones, we analysed scaled values of cumulative bone apposition areas. A scaled value is a measure of how many standard deviations a raw score is below or above the mean of all measurements of a particular bone. Therefore, scaled values have a mean of zero and include equal amounts of negative and positive deviations in the case of a symmetric distribution. Raw data (i.e. without scaling) can be found in Fig. SM11, SM12 and Tables SM1 and SM4. We considered repeated measurements in linear mixed effects models by a random factor bone nested within individuals.

## Supplementary Information

Supplementary Information 1.
