# Supplementary material for: Labelling experiments in red deer provide a general model for early bone growth dynamics in ruminants
Source: Sci Rep. 2021 Jul 7;11:14074. doi: 10.1038/s41598-021-93547-4 (PMC8263734; doi:10.1038/s41598-021-93547-4)
Supplement: Supplementary file 1 — Supplementary Information 1. [file 41598_2021_93547_MOESM1_ESM.pdf]

Supplementary material for:

Labelling experiments in red deer provide a general model for early bone growth dynamics in ruminants

Teresa Calderón[a]*, Walter Arnold[b], Gabrielle Stalder[b], Johanna Painer[b], Meike Köhler[a,c]

[a] Institut Català de Paleontologia Miquel Crusafont (ICP). Edifici Z, C/ de les Columnes, s/n., Campus de la Universidad Autònoma de Barcelona, 08193 Bellaterra, Barcelona (Spain).

[b] Wildlife Medicine and Applied Ecology, Research Institute of Wildlife Ecology, University of Veterinary Medicine (Vetmeduni Wien), Vienna (Austria).

[c] ICREA, Pg. Lluís Companys 23, 08010 Barcelona (Spain).

DETAILED HISTOLOGICAL DESCRIPTION

Perinatal growth

Femur

Few days after birth, the cross-section is rounded with a narrow compact bone bordering a large medullary cavity. Most of the bone cortex, composed of primary bone, has been formed before birth, resulting in a disorganized matrix of prenatal fibro-lamellar complex (FLC) with a high amount of parallel-fibered bone (PFB). The tissue within the prenatal tissue is mainly reticular except for the postero-medial and, inversely, for the antero-lateral regions. In these areas the bone tissue shows plexiform and longitudinal arrangments, respectively. After birth, the femur area increases towards the antero-medial part. Postnatal tissue is barely deposited in the postero-medial sector of the bone. In the subsequent days FLC is deposited following a pattern of plexiform orientation.

Humerus

A narrow bone cortex of mainly disorganized prenatal tissue with high proportion of primary PFB (Suppl. Fig. 1a), encloses a large medullary cavity. The tissue shifts from reticular to a plexiform/reticular in the medial and lateral sectors during prenatal growth and barely increases in area after birth. The small area of postnatal tissue is deposited towards the posterior part.

Tibia

At this early age, the bone shaft is quite rounded and is almost entirely composed of prenatal tissue with FLC matrix. In the prenatal area, the FLC alternates between reticular and plexiform, showing a more organized pattern towards the outer part of the antero-medial sector of the bone. Some dispersed radial canals are discernible in the lateral part (Suppl. Fig. 1b). The cortical apposition of postnatal bone is arranged asymmetrically. Abundant bone is deposited towards the lateral portion. At the opposite site, the postnatal tissue forms an extremely thin band. The FLC in this area is plexiform/reticular.

Radius

The cross-section of this bone shows an elongated shape with considerable cortical thickness along the antero-posterior axis. Most of the cortex is composed of prenatal tissue. In the inner part of the medial portion the bone tissue is reticular. Afterwards, as

FLC increases in area, the FLC becomes plexiform. The rest of the bone shows FLC with plexiform/reticular arrangement except for the postero-lateral sector, which presents plexiform/laminar arrangement. Postnatal bone is deposited only anterior and posteriorly with a plexiform/reticular pattern. The endosteal bone starts to deposit at the third day of life both in the anterior and posterior areas of the shaft. This tissue is separated from the primary bone by a cement line. A scaffold of woven bone forms this new tissue centripetally.

Metapodial bones

Metapodials are characterized by a rounded shape of two lobes separated from each other by a column in the centre of the medullary cavity. Most of the cortex is formed by prenatal reticular FLC that locally shifts to a plexiform as new bone is deposited. There is only little postnatal apposition towards the lateral side. In both metapodials, the endosteal bone starts to deposit on the 3rd day in the anterior half of the bone. From here on, a scaffold of woven bone is deposited centripetally thus reducing the area of the two semi-medullar cavities. In the metatarsus, a thin layer of lamellar bone precedes the woven scaffold.

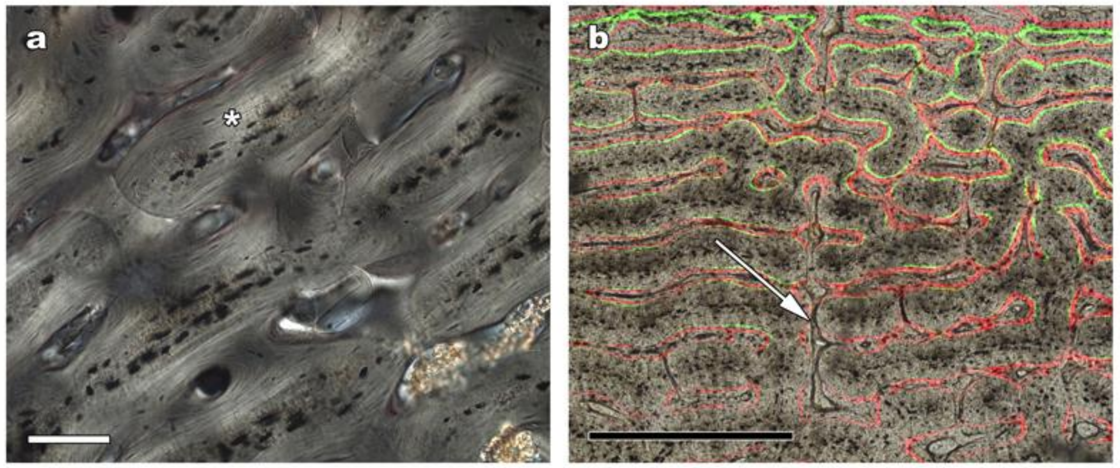

Supplementary Figure 1. Histological details of the 15 days old individual ID-1 / IPS-88714 under polarized light (a) and fluorescent light (b). Images are oriented with the medullary cavity (MC) towards the inferior part and outermost cortex (OC) towards the superior side unless specified. (a) High proportion of PFB (asterisk) in the prenatal part of the humerus. (b) Detail of the radial canals (white arrow) in the prenatal tissue of the tibia. Black scale: 500 micrometres. White scale: 100 micrometres.

Growth before weaning

Femur

At the age of 15 weeks, the shape of the cross section still is quite rounded, but the linea aspera is identifiable in the postero-medial sector. At this stage, the area of postnatal tissue increases while the prenatal area has been eroded by the expansion of the medullary cavity (Suppl. Fig. 2a). This expansion is more pronounced in the postero-medial sector, where the tissue formed before the third day of life has been partially deleted. As the area of postnatal tissue increases, the FLC becomes somewhat more organized: from an early plexiform/reticular orientation (from 3 to 30 days of life) to a plexiform or even plexiform/laminar organisation in the outer cortex (from 30 days to 15 weeks). In addition to this shift, tissue shows a compressed arrangement between days 3 and 30. The short distance between labels in the medial area reveals the slow growth of the tissue in this sector. There is scarce deposition of tissue from weeks 13 to 15 in all sectors. At the age of 15 weeks there is no formation of new scaffold, but tissue is deposited within the scaffold previously formed. In general, the pattern is more organized towards the medial and posterior sectors, except for the linea aspera, which shows a reticular orientation. In this area, a few cavities formed at 13 weeks are refilled with secondary bone in the two subsequent weeks.

Humerus

The prenatal tissue is practically non-existent in the posterior and postero-lateral areas due to the considerable directional expansion of the medullary cavity. There is a greater amount of cortical apposition towards the posterior and medial sectors than in the rest of the bone, resulting in a wider separation of the labels. By contrast, labels are closer spaced within the antero-medial region. Bone tissue shows a great variability of FLC types: longitudinal in the antero-lateral part, plexiform in the posterior one and a band of laminar oriented canals is deposited in the outer part of the lateral and medial sectors. As occurs in the femur, bone is deposited in a more compact manner during the interval between 3 and 30 days of life than in the other intervals. There is no increase of bone area in the lateral portions between weeks 13 to 15. However tissue is deposited within the inner cavities of the previously formed scaffold. At this age, an incipient endosteal bone composed of lamellar tissue is deposited in the antero-medial region.

Tibia

The expansion of the medullary cavity towards the lateral part has partially eroded the perinatal bone. The labels evidence increased bone apposition on the lateral side. As a result, the proportion of pre- and postnatal tissue varies over the shaft. In the antero-medial portion, the prenatal tissue formed by reticular FLC still predominates in most of the area, while the lateral part is almost entirely formed by postnatal tissue. The organisation of the postnatal FLC varies depending on the bone area. Within the lateral portion of the shaft a significant amount of plexiform tissue has been deposited between the labels. The interval from 3-30 days shows higher compactness than the rest of the deposition. There is a marked bone drift in the anterior and medial parts of the tibia between pre- and postnatal tissue. In a small portion of these anterior and medial areas, bone deposited between 3 and 30 days has been resorbed, leaving a scalloped surface delimited by a line (these lines are associated to particular areas that show cortical drift and sometimes bone resorption; Suppl. Fig. 2b). Newly deposited tissue shows a laminar osteonal orientation with a lower degree of compactness than in the rest of the bone (Suppl. Fig. 2c). Bone apposition between weeks 13 to 15 is only observable in the lateral area of the tibia (i.e. new scaffold is formed at this age). At this stage secondary bone starts to form. A scaffold of woven bone builds the endosteal bone and incipient Haversian systems appear in the antero-medial sector. According to the labels, both structures started to form some time before the age of 13 weeks and began to refill until the death of the individual after the 15th week.

Radius

The expansion of the medullary cavity through the anterior and posterior areas has deleted a small portion of prenatal tissue and the endosteal bone deposited at previous stages. Cortical apposition of bone also follows this asymmetrical trend. At the age of 3 days, there is no periosteal growth in antero-posterior direction. Bone is deposited within the cavities of the prenatal scaffold and also within the annulus that delimitates the prenatal tissue (Suupl. Fig. 2d). The subsequent deposition of FLC follows a plexiform/reticular arrangment, but between days 3 to 30, bone is deposited with a higher degree of compactness than in previous and posterior intervals. Moreover, an area of reticular bone appears in the posterior sector. In a small section of the medial part the tissue deposited between 3 and 30 days has been resorbed and a line limits the area. Afterwards, FLC is deposited following laminar orientation. There is no increase of bone area in the lateral portions between weeks 13 to 15. At the age of 30 days, the endosteal bone starts to form at the medial side of the radius. This structure is formed by longitudinal and reticular FLC with some areas of highly organized and

non-vascular tissue. Incipient Haversian systems appear in the medial part of the prenatal tissue. Cavities formed at 13 weeks are refilled with new tissue in the subsequent weeks.

Metapodial bones

At this stage, the expansion of the medullary cavity has removed completely the central column of the metatarsus, although this structure is still visible in the metacarpus. The postnatal tissue is deposited mainly towards the posterior sector both in metacarpus and metatarsus. The principal tissue fund is reticular FLC, with some small plexiform/laminar areas in the anterior and posterior sectors. The label arrangement emphasises the scarce lateral and medial tissue apposition between days 3 and 30. In the postero-lateral sector of the metatarsus, the tissue formed during this interval has been resorbed and a line can be distinguished (Suppl. Fig. 2e). A broad band rich in lamellar tissue appears along the outer part of the line. There is hardly bone apposition between 13-15 weeks. At this age, bone is only deposited within the cavities left by the previous scaffold. The endosteal bone, still present since its early formation, continued refilling the woven bone matrix following a longitudinal pattern throughout the cortex but the posterior sector (Suppl. Fig. 2f). In the postero-lateral and the posterior-medial sectors of both metapodials, Haversian systems started to form at 13 weeks by actively refilling the cavities at the age of 15 weeks.

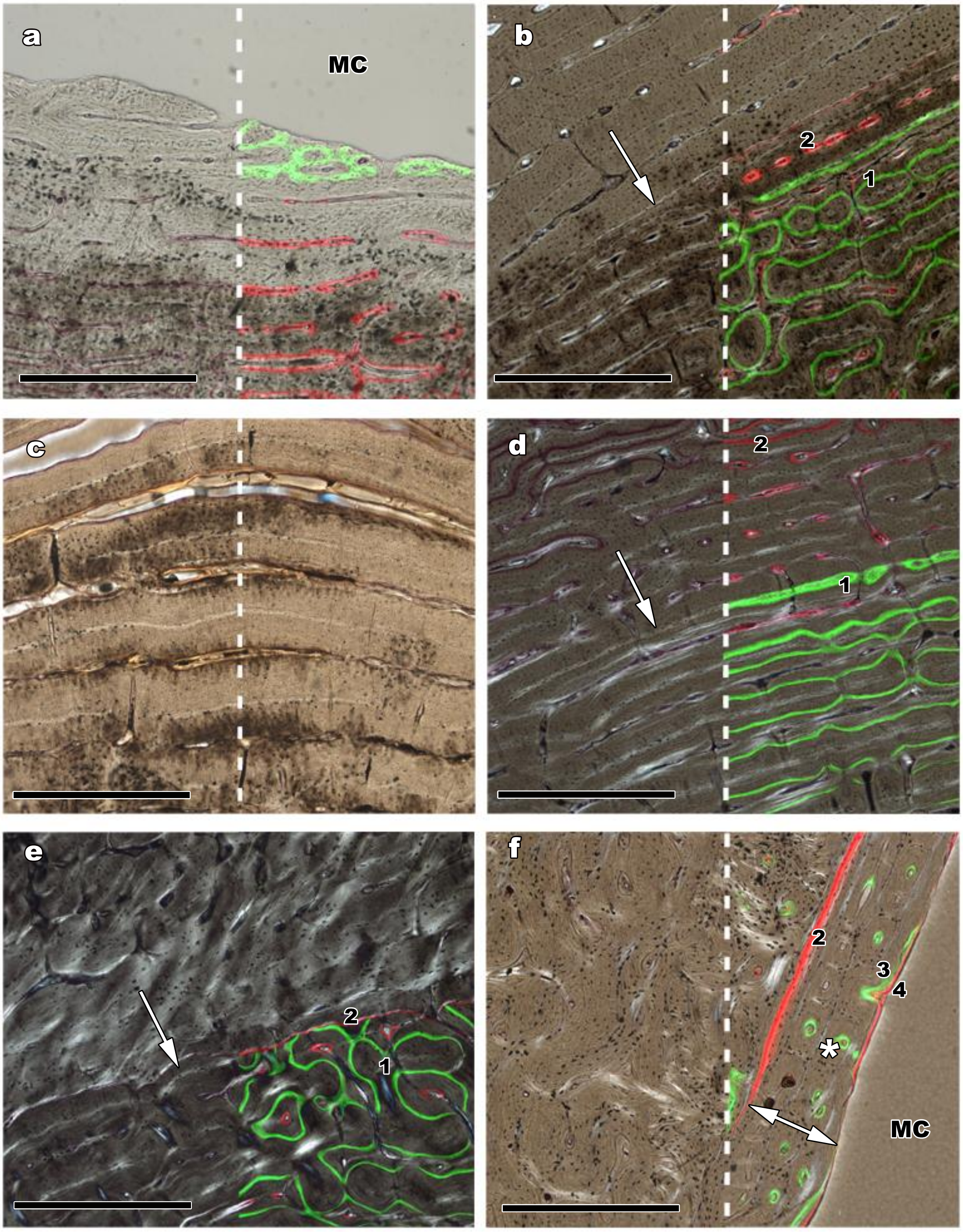

Supplementary Figure 2. Histological detail of the 15 weeks old individual ID-2 / IPS-88715 under combined transmitted and fluorescent light (a), polarized light (c) and combined polarized and fluorescent light (d, e, f). Images are oriented with the medullary cavity (MC) towards the inferior part and outermost cortex (OC) towards the superior side unless specified. (a) Partial erosion of prenatal bone my medullary expansion. (b) LAG indicated by white arrow delimiting the resorbed cortex in the anterior sector of the tibia. (c) Laminar FLC in the antero-medial part of the tibia. (d) Annulus bordering the prenatal growth (white arrow) in the posterior part of the radius

(e) Scalloped surface left by a resorption process in the postero-lateral sector of the metatarsus. (f) Endosteal bone in the lateral sector of the metatarsus (white arrow) composed of longitudinal osteons (asterisk). 1: label deposited at 3 days; 2: label deposited at 30 days; 3: label deposited at 13 weeks; 4: label deposited at 15 weeks. Black scale: 500 micrometres.

Growth after weaning

Femur *(hand-raised individuals)*

At this stage, the prenatal tissue consists of a narrow band around the medullary cavity, which is slightly broader in the medial area. Despite, individuals ID-3 and ID-4 were not labelled during the perinatal growth, the shift from pre- to postnatal tissue is identifiable due to the different proportion of primary PFB within the FLC and the presence of an annulus surrounding part of the prenatal area in the case of the male ID-3. Postnatal tissue, composed of FLC, predominates within the bone section. As mentioned for the previous stage, we also observe a more organized pattern towards the posterior part or the bone. The labels of 13, 15 and 17 weeks are closely deposited throughout the entire shaft, indicating a limited bone apposition during this period. Indeed, there is almost no periosteal growth in some sections of the bone. Instead, there are partial LAGs observable between the weeks 13 to 17 in the anterior parts of male ID-3 and female ID-4. In these areas, there is no increase in bone area between weeks 13 to 15, but tissue is refilling the previously formed scaffold. The green-labelled surface deposited at 17 weeks appears as a narrow line bordering the outer part of the LAG, indicating that bone apposition stopped for almost one month after weaning in these areas and restarted at week 17. Before and after this LAG, there are different types of FLC (Suppl. Fig. 3a). Thus, a plexiform/reticular FLC shifts to plexiform/laminar in the anterior portion of the femur and from plexiform to laminar towards the posterior part. Endosteal bone is located in a small area of the lateral and anterior part of ID-3 and ID-4, respectively. This structure is separated from the primary bone by a cement line. In this tissue, a plexiform arrangement of osteons prevails. The Haversian system can be found in area of the linea aspera and incipiently in the prenatal area, eroding the primary tissue. Some cavities are still forming in this area.

Femur *(individuals nursed by mother)*

In the two individuals analysed, the proportion of pre- and postnatal bone are the same as in hand-raised individuals, as well as the organisation of the endosteal bone and

Haversian systems. Femora of these individuals, however, show a more organized pattern in the tissue of the posterior sector. These bones are composed of disorganized and reticular areas at the beginning of the postnatal deposition; thereafter, they show a constant deposition of plexiform FLC without any shift or disruption. In both individuals, ID-23 and ID-24, the endosteal bone is an only a thin layer.

Humerus *(hand-raised individuals)*

The section of the humerus shows a pronounced and directional expansion of the medullary cavity and bone apposition towards the postero-medial sector. The significant increase of the medullary cavity in these areas deletes almost all tissue deposited before 13 weeks. In the opposite section, the prenatal tissue is still preserved and it is even possible to distinguish a partial annulus at the moment of birth. The tissue of the medial sector is reticular, while it is plexiform in the posterior sector (slightly more organized in the female individual ID-4). Plexiform FLC is deposited at the lateral side of the bone until week 13. Labels deposited between weeks 13 to 17 are narrowly spaced throughout the entire section with exception of the posterior portion, where they are wider spaced, which denotes the scarce tissue deposition along most of the periosteum during this interval. Actually, in the antero-lateral sector of both individuals (ID-4 and ID-3) there is no periosteal apposition for two weeks (13 to 15); however, bone continues to be deposited within the previously formed scaffold. A partial LAG can be identified between this scaffold and the subsequently formed new laminar bone shortly before week 17. There is a thick layer of endosteal bone enclosed within a small area of the anterior sector formed some time before week 13. At its beginning, the endosteal bone consists of lamellar tissue, but later in ontogeny the patterns shifts to plexiform. The Haversian systems appear enclosed within a small area in the medial sector of the humerus.

Humerus *(individuals nursed by mother)*

As well as in the hand-raised group, the humerus of individuals nursed by mother shows an area of reticular FLC in the medial sector, while plexiform tissue predominates in the rest of the section. None of the individuals shows a shift in bone tissue after 13 weeks. As well as in the femora, the endosteal bone forms a thin layer bordering the medullary cavity.

Tibia *(hand-raised individuals)*

The tibia is mainly composed of postnatal tissue at this stage, though there still remains a well distinguishable residual prenatal area surrounded by an annulus or a partial LAG in the posterior area of both individuals ID-3 and ID-4 (Suppl. Fig. 3b). The postnatal bone tissue is plexiform/laminar during the first 13 weeks. We observe a marked bone drift in the anterior part of the tibia. In this area, cortical resorption has eroded parts of the tissue deposited between weeks 13 to 17, leaving a scalloped edge limited by a line. Between 17 and 23 weeks, tissue deposition resumes but it follows a different osteon arrangement. Radial canals are distributed within the entire cross-section, being the highest densities of these canals in the postero-lateral region. The Haversian systems spread almost throughout the entire prenatal area and new cavities are still forming. The expansion of the medullary cavity erodes the endosteal bone formed at previous stages and a small area of new endosteal tissue starts to form around the 13th week following reticular and longitudinal orientation.

Tibia *(individuals nursed by mother)*

This bone is composed of a plexiform FLC, with the medial part slightly more organized than the lateral one. The prenatal tissue is bordered by a partial LAG in the anterior sector of the ID-23 tibia. The medial sector shows a small area of reticular bone in ID-24 after the 13th week. The endosteal bone has a longitudinal orientation while in ID-23 a plexiform FLC alternates with lamellar bone.

Radius *(hand-raised individuals)*

At this stage, this bone shows a thick cortex and a small medullary cavity. As a result, the prenatal tissue still occupies a considerable area within the cross-section, delimited by an annulus in the medial sector. As a particularity, the early postnatal tissue of ID-3 shows some longitudinal osteons in the anterior sector. From now onwards until week 13, cortical apposition follows the plexiform FLC described previously, with dispersed reticular areas in the anterior and posterior sectors. Towards the medial and lateral sides of both individuals, a resorption process has eroded part of the tissue deposited between weeks 13 and 17 (Suppl. Fig. 3c). As a result these areas show well-marked lines and an abrupt shift in orientation. Some lines form in the lateral part in subsequent post-weaning weeks; endosteal bone is reticular, plexiform and laminar. Well-developed Haversian systems can be found within the almost entire prenatal tissue. They also spread within the lateral part of the bone.

Radius *(individuals nursed by mother)*

At the beginning of the postnatal deposition, bone shows a plexiform FLC that shifts rapidly to a reticular, which prevails after weaning. Tissue deposited at 13 weeks is resorbed in subsequent stages as testify the eroded label and a line in the medial and lateral sectors of ID-24 (Suppl. Fig. 3d). These structures are also visible in the medial and lateral part of ID-23 shortly before and after week 13. The endosteal tissue in this bone is formed by FLC with plexiform and laminar orientation in ID-23 and longitudinal orientation in ID-24.

Metapodial bones *(hand-raised individuals)*

The medullary cavity shows a reduced size at this stage, but their expansion has completely removed the central column in both bones. The prenatal bone is visible within most of the bone shaft but in the posterior sector, being delimited by a partial LAG in the ID-3 metatarsus. As in the radius, metatarsus and metacarpus of ID-3 show a band of longitudinal osteons just at the beginning of the postnatal deposition in the antero-lateral part of the bone (Suppl. Fig. 3e). In general, during the first 13 weeks bone matrix is deposited following a continuous reticular FLC. Labels from 13 to 17 weeks are closely spaced except for the posterior area, which indicates a slower tissue apposition during this period. Indeed, in the anterior sector a partial LAG represents an arrest of growth between weeks 15 and 17. At the inner side of this LAG there is a thin layer of bone deposited at week 15, while bone deposited at week 17 surrounds the LAG externally. The tissue deposited at week 17 shows plexiform/laminar FLC. In the medial sector of ID-4 metapodials, a band of laminar bone appears even after the 13th week. A broad band of endosteal bone is present in the anterior sector of the bones (Suppl. Fig. 3f), occupying a comparatively larger area in the metacarpus. The Haversian systems are limited to the postero-lateral and postero-medial regions, though large erosion cavities penetrate throughout the whole prenatal tissue zone.

Metapodial bones *(individuals nursed by mother)*

At the age of six months, there still remains a part of the central column in the medullary cavity of ID-24 metacarpus but not in that of ID-23. The main FLC pattern in these bones is uniformly reticular, although there is a more organized arrangement, even plexiform, towards the medial and lateral sides. In the medial part of the ID-24 metacarpus, a partial LAG delimitates the prenatal tissue. The endosteal bone in the metatarsus is composed by reticular and plexiform FLC, while the metacarpus shows a longitudinal pattern.

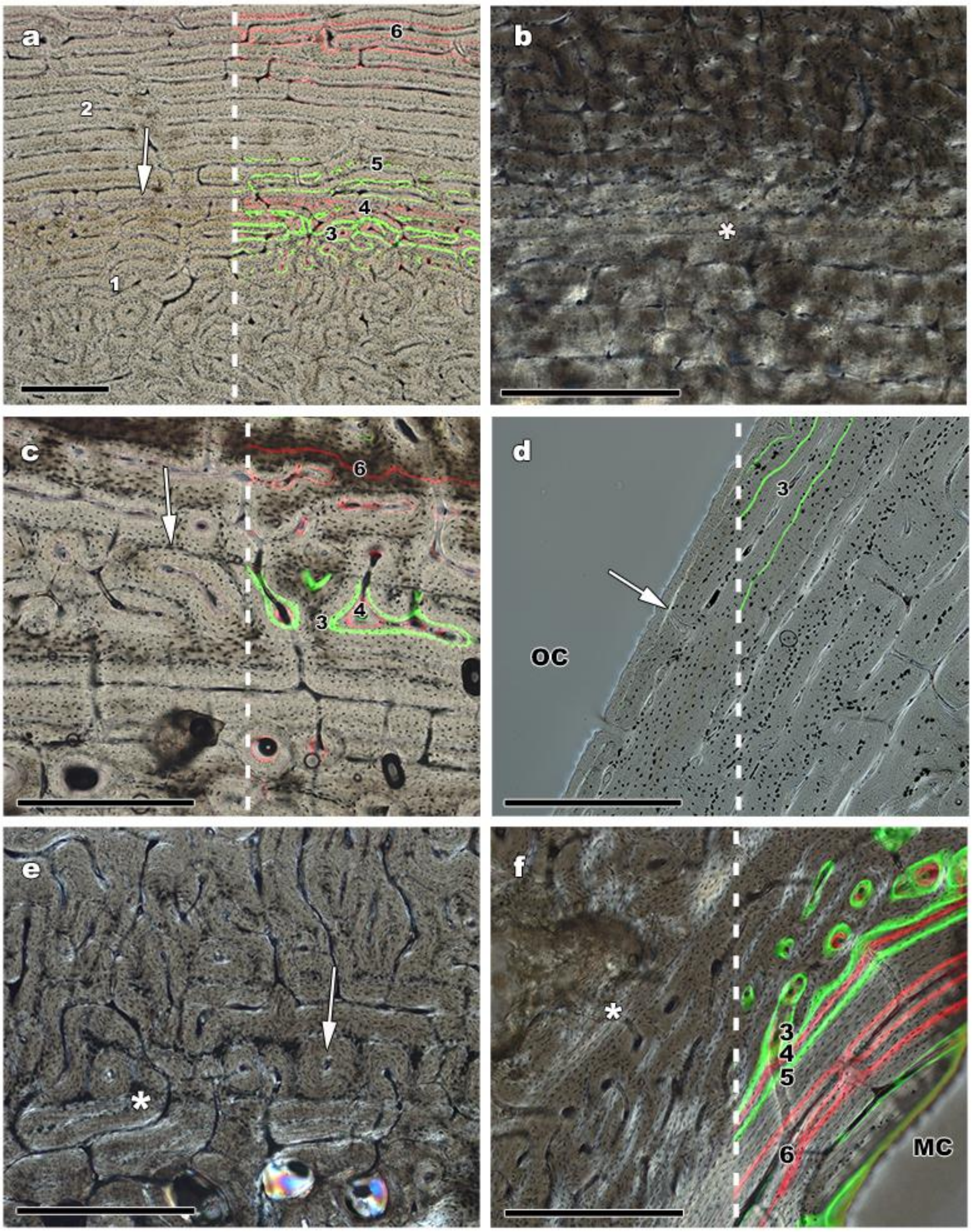

Supplementary Figure 3. Histological detail of individuals older than 15 weeks under combined polarized and fluorescent light (a, c, d, f) and polarized light (b,e). Images are oriented with the medullary cavity (MC) towards the inferior part and outermost cortex (OC) towards the superior side unless specified. (a) Shift in the orientation of osteons in the anterior part of the ID-3 / IPS-93664 femur separated by a partial LAG (white arrow). Number 1 written in white indicates reticular deposition; number 2 written in white indicates plexiform orientation deposited from the week 17 onwards. (b) LAG bordering the prenatal tissue (white asterisk) in the posterior part of the ID-3 / IPS-

93664 tibia. (c) LAG (white arrow) in the medial part of ID-3 / IPS-93664 radius indicating the interruption of tissue deposition over six weeks. (d) LAG (white arrow) deposited at the age of 13 weeks in the antero-lateral sector of ID-24 / IPS-109291 radius. (e) Longitudinal osteons at the beginning of postnatal deposition (white arrow) in the anterior sector of ID-3 / IPS-93664 metatarsus. The white asterisk marks the border of prenatal tissue. (f) Endosteal bone in the antero-lateral sector of ID-3 / IPS-93664 metatarsus; the cement line is marked with an asterisk. 3: label deposited at 13 weeks; 4: label deposited at 15 weeks; 5 label deposited at 17 weeks; 6: label deposited at 23 weeks. Black scale: 500 micrometres.

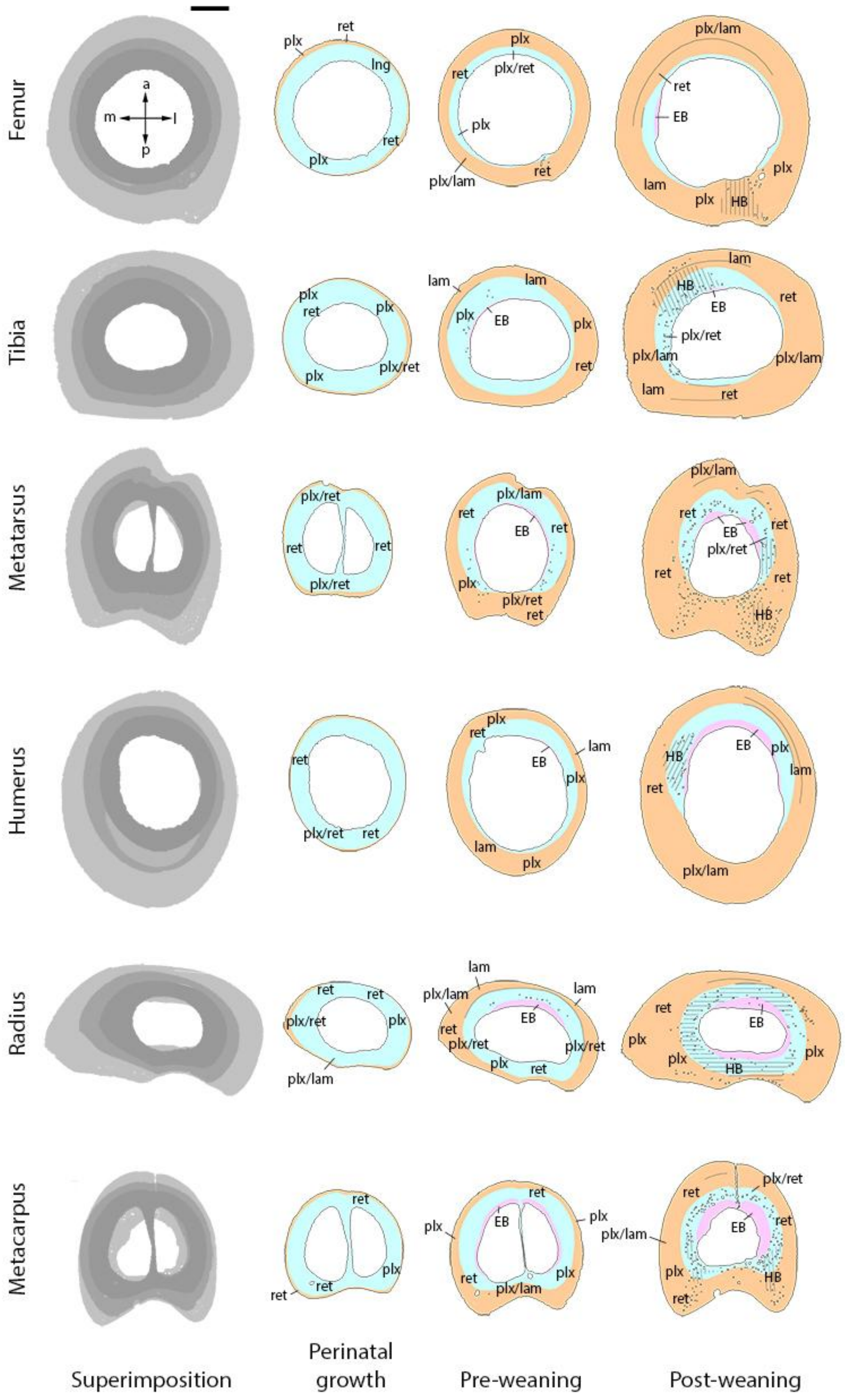
Femur
Tibia
Metatarsus
Humerus
Radius
Metacarpus
a
m
l
p
Superimposition
Perinatal growth
Pre-weaning
Post-weaning

Supplementary Figure 4. Superimposed outline of bone shaft sections and chronologically ordered by age to schematically illustrate perinatal growth (ID-1 / IPS-88714), growth during the pre-weaning period (ID-2 / IPS-88715) and growth after weaning (ID-3 / IPS-93664). Scale: 5mm. Dark grey: perinatal stage; Intermediate grey: pre-weaning stage; Light grey: post-weaning stage; Blue color: prenatal tissue (primary bone); orange color: postnatal tissue (primary bone); pink color: endosteal bone (secondary bone); dashed area: haversian bone (secondary bone); lam: laminar; plx: plexiform; ret: reticular; HB: Haversian bone; EB: Endosteal bone. A: anterior; P: posterior; M: medial; L: lateral.

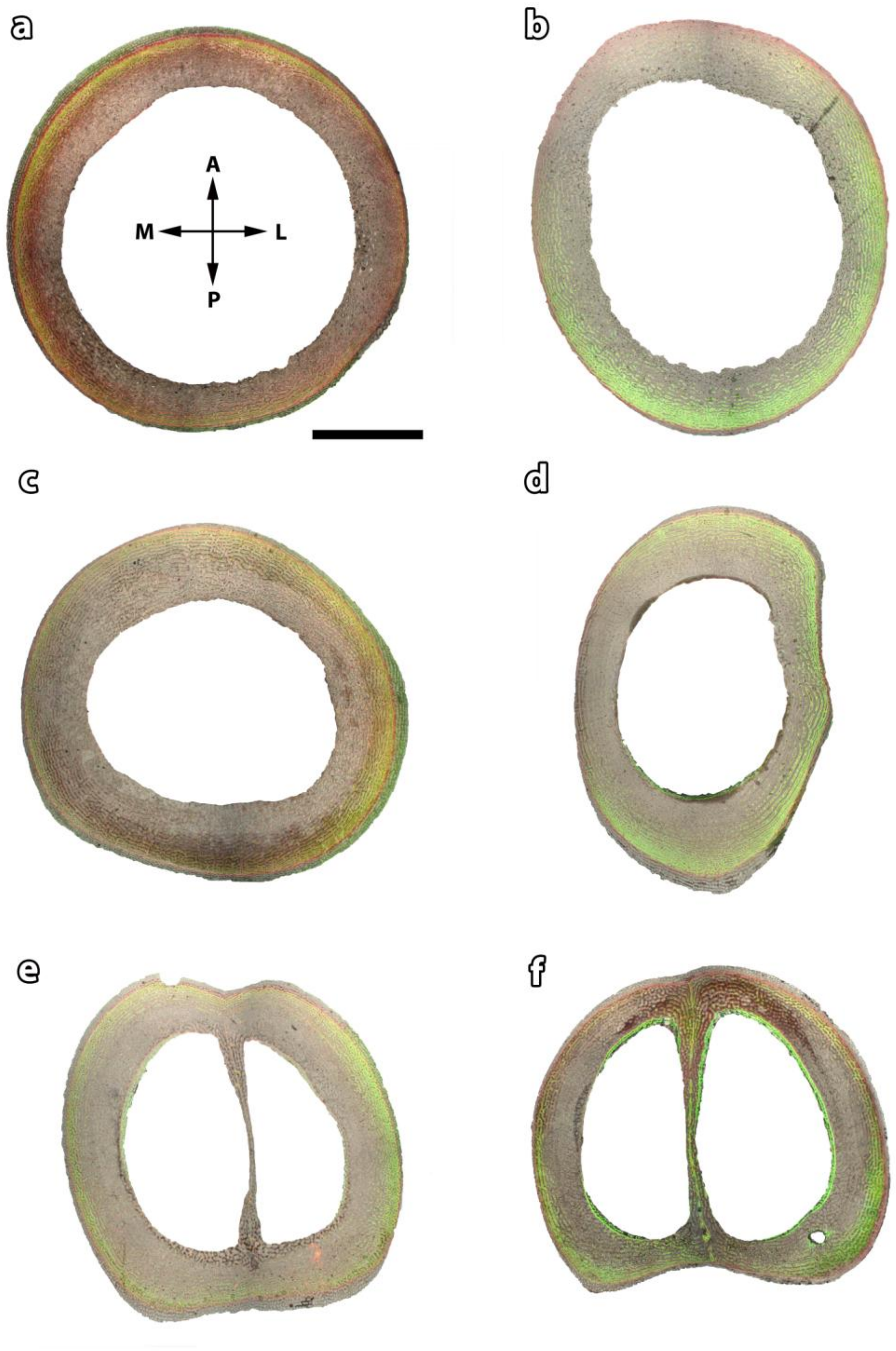

Supplementary Figure 5. Histological compositions of the hindlimb (left) and forelimb (right) bones of the 15 days old individual ID-1 / IPS-8814 labelled at birth, and days 3 and 15. (a) femur. (b) humerus. (c) tibia. (d) radius. (e) metatarsus. (f) metacarpus. Scale: 5 milimetres.

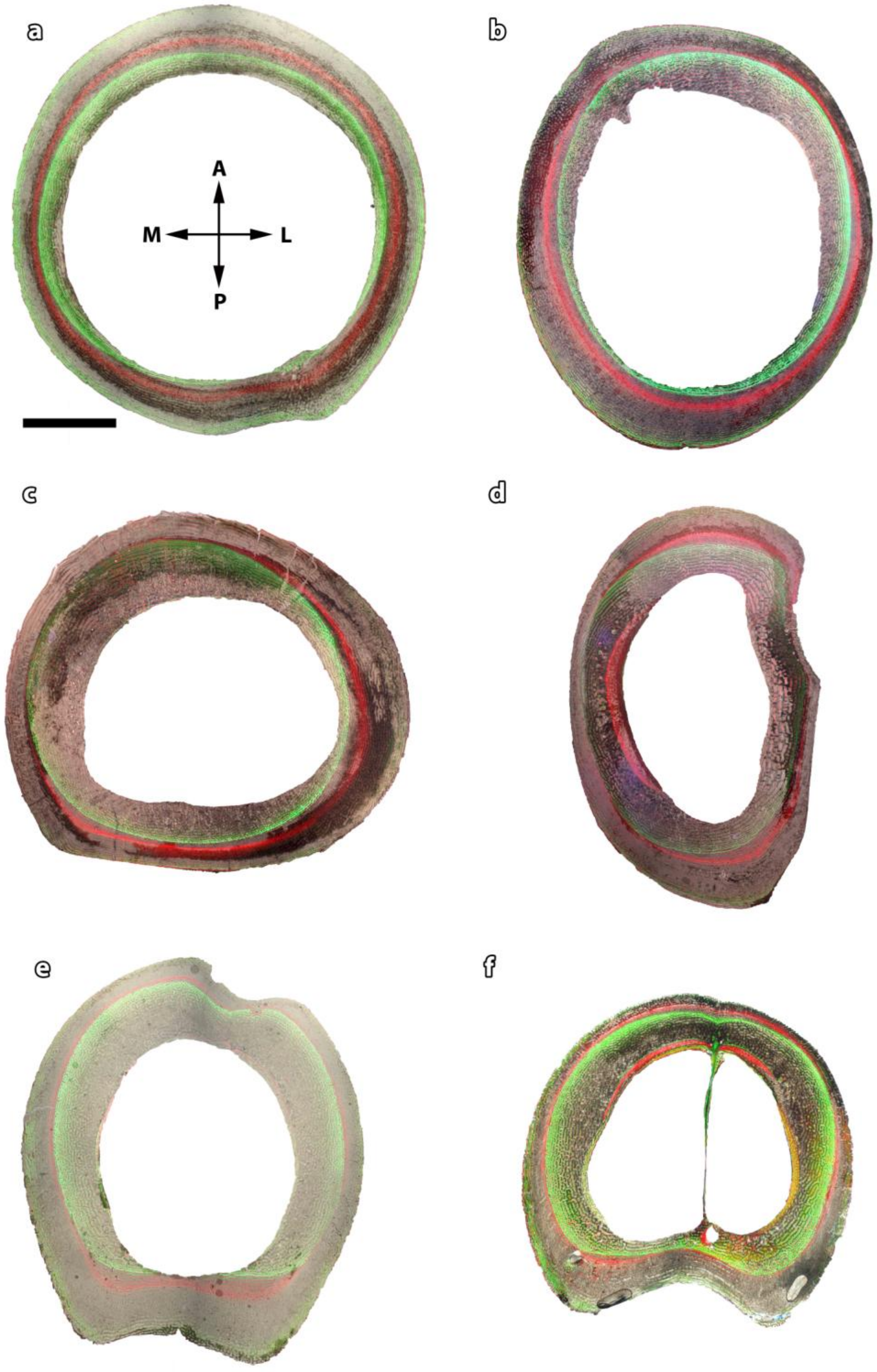

Supplementary Figure 6. Histological compositions of the hindlimb (left) and forelimb (right) bones of the 15 weeks old individual ID-2 / IPS-8815 labelled at days 3 and 30, and weeks 13 and 15. (a) femur. (b) humerus. (c) tibia. (d) radius. (e) metatarsus. (f) metacarpus. Scale: 5 milimetres.

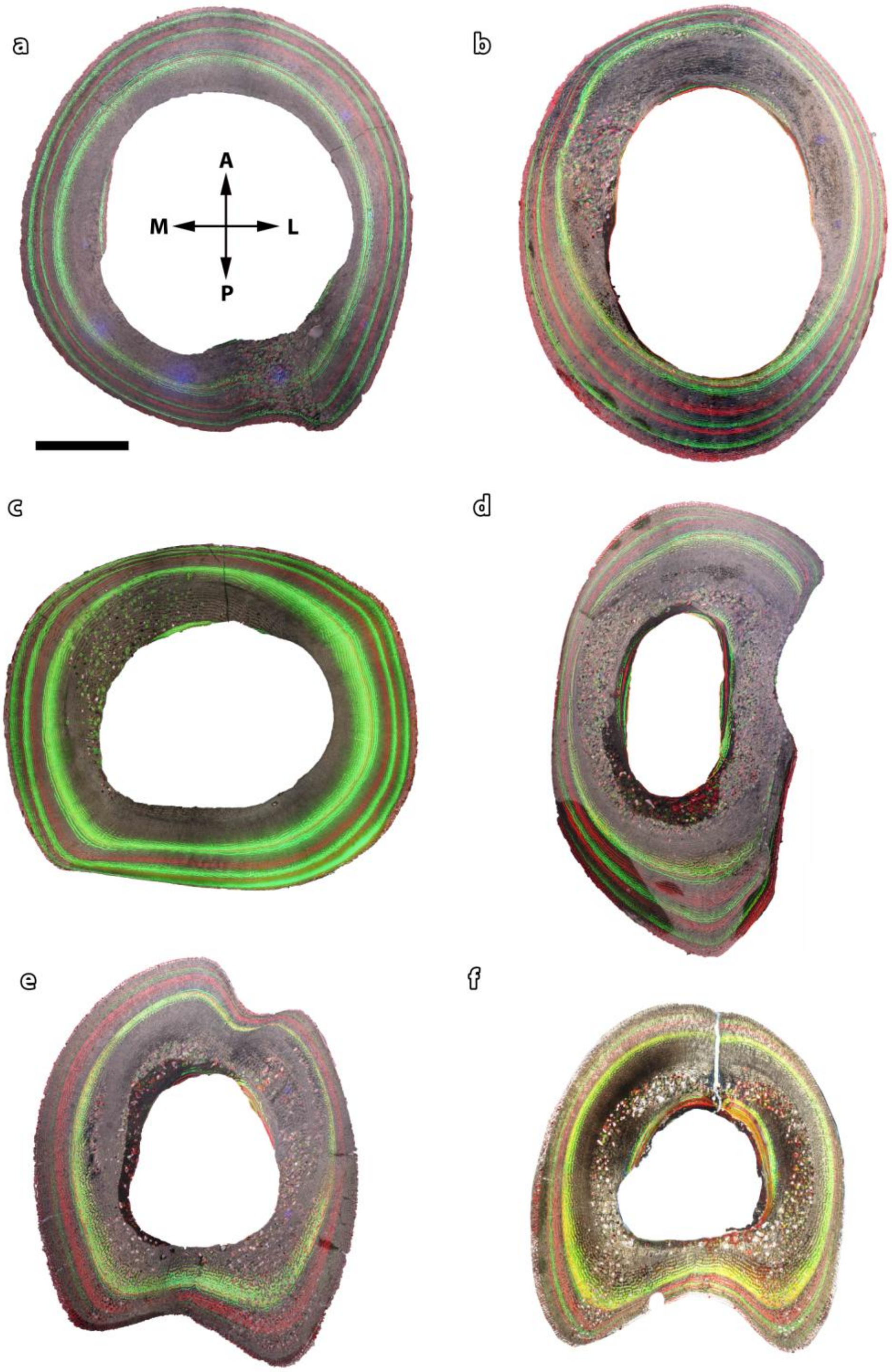

Supplementary Figure 7. Histological compositions of the hindlimb (left) and forelimb (right) bones of the 43 weeks old individual ID-3 / IPS-93664 labelled at weeks 13, 15, 17 and 23 (subsequent labels have not been used in this study) (a) femur. (b) humerus. (c) tibia. (d) radius. (e) metatarsus. (f) metacarpus. Scale: 5 milimetres.

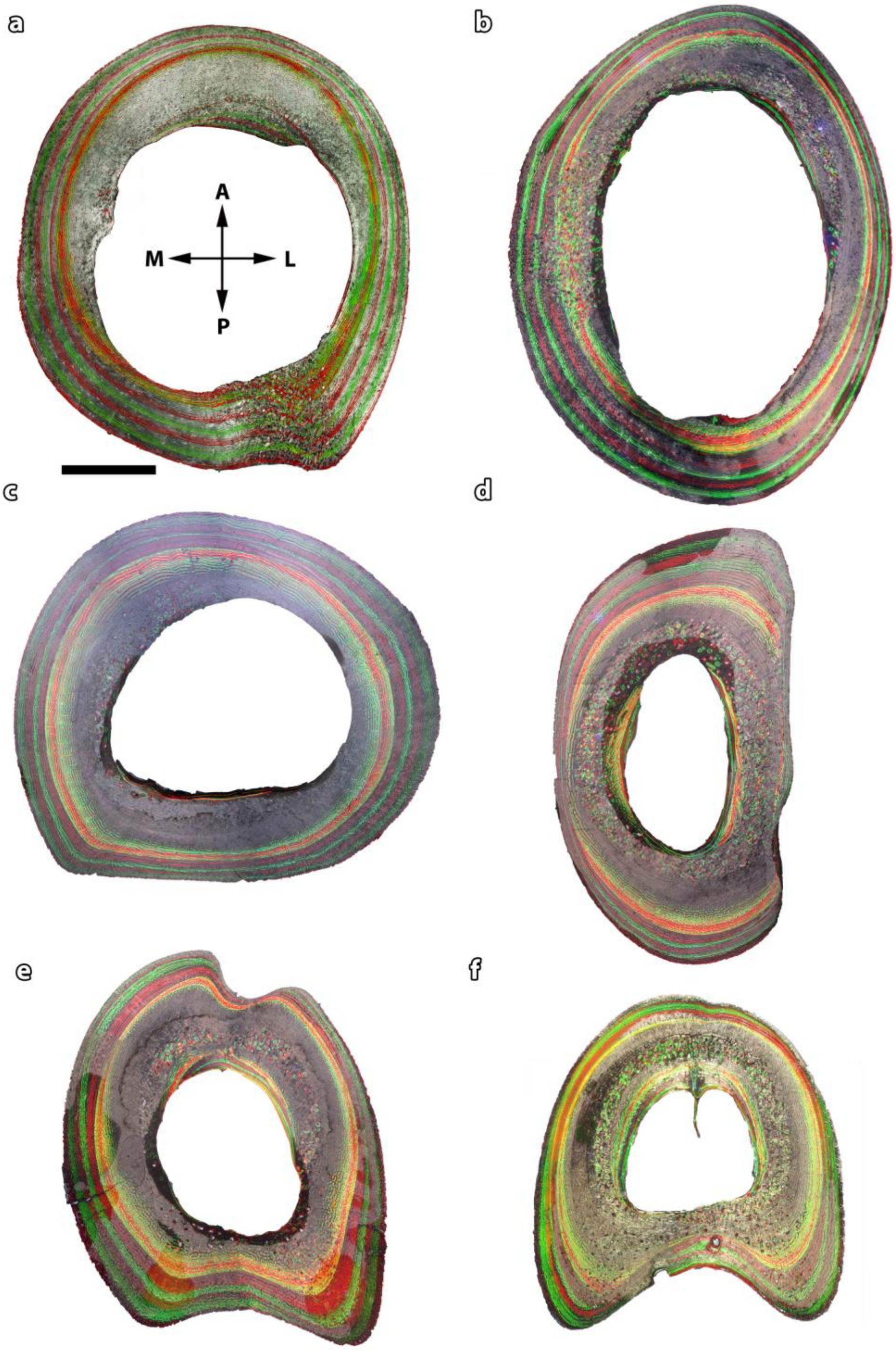

Supplementary Figure 8. Histological compositions of the hindlimb (left) and forelimb (right) bones of the 43 weeks old individual ID-4 / IPS-88713 labelled at weeks 9, 11,13, 15, 17 and 23 (subsequent labels have not been used in this study) (a) femur. (b) humerus. (c) tibia. (d) radius. (e) metatarsus. (f) metacarpus. Scale: 5 milimetres

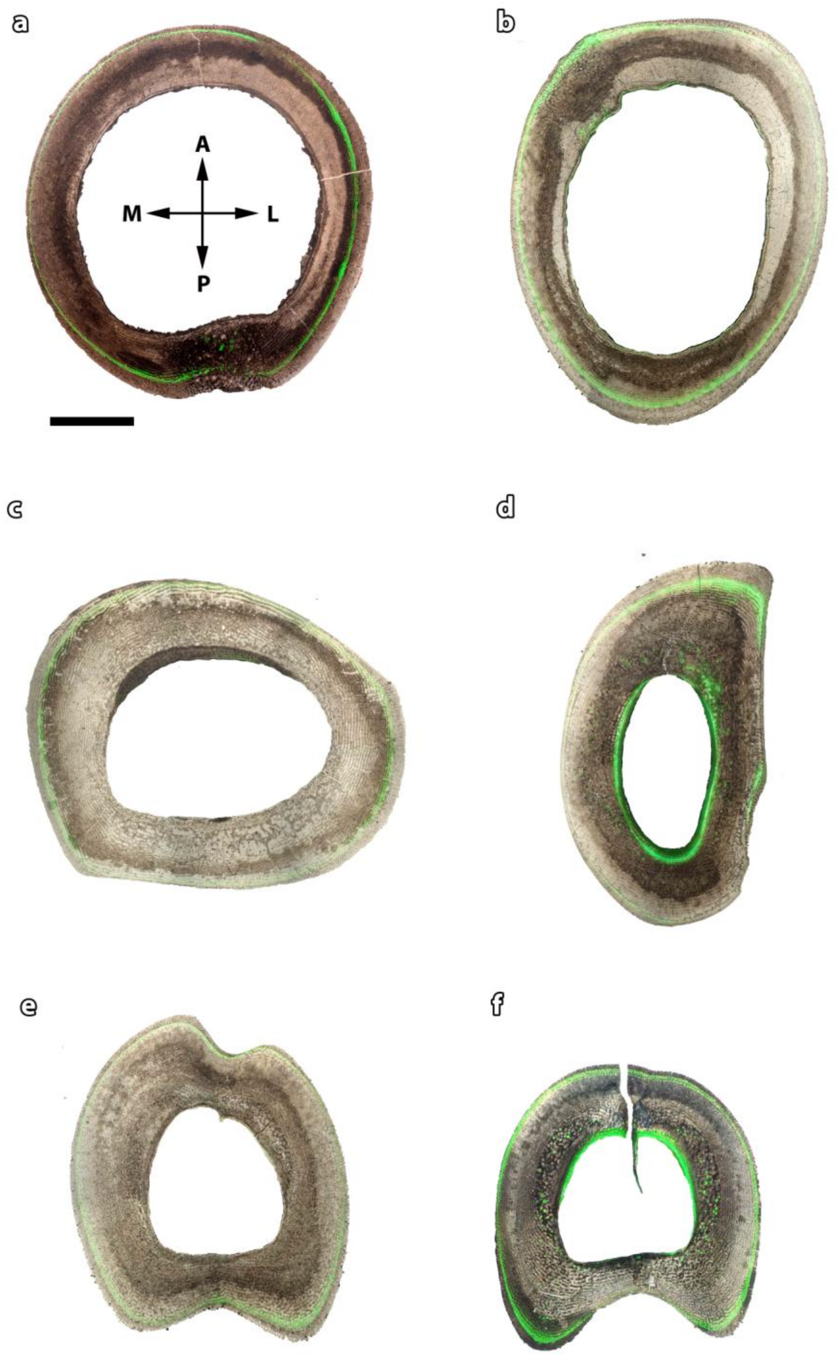

Supplementary Figure 9. Histological compositions of the hindlimb (left) and forelimb (right) bones of the 24 weeks old individual ID-24 / IPS-190291 labelled at week 13. (a) femur. (b) humerus. (c) tibia. (d) radius. (e) metatarsus. (f) metacarpus. Scale: 5 milimetres

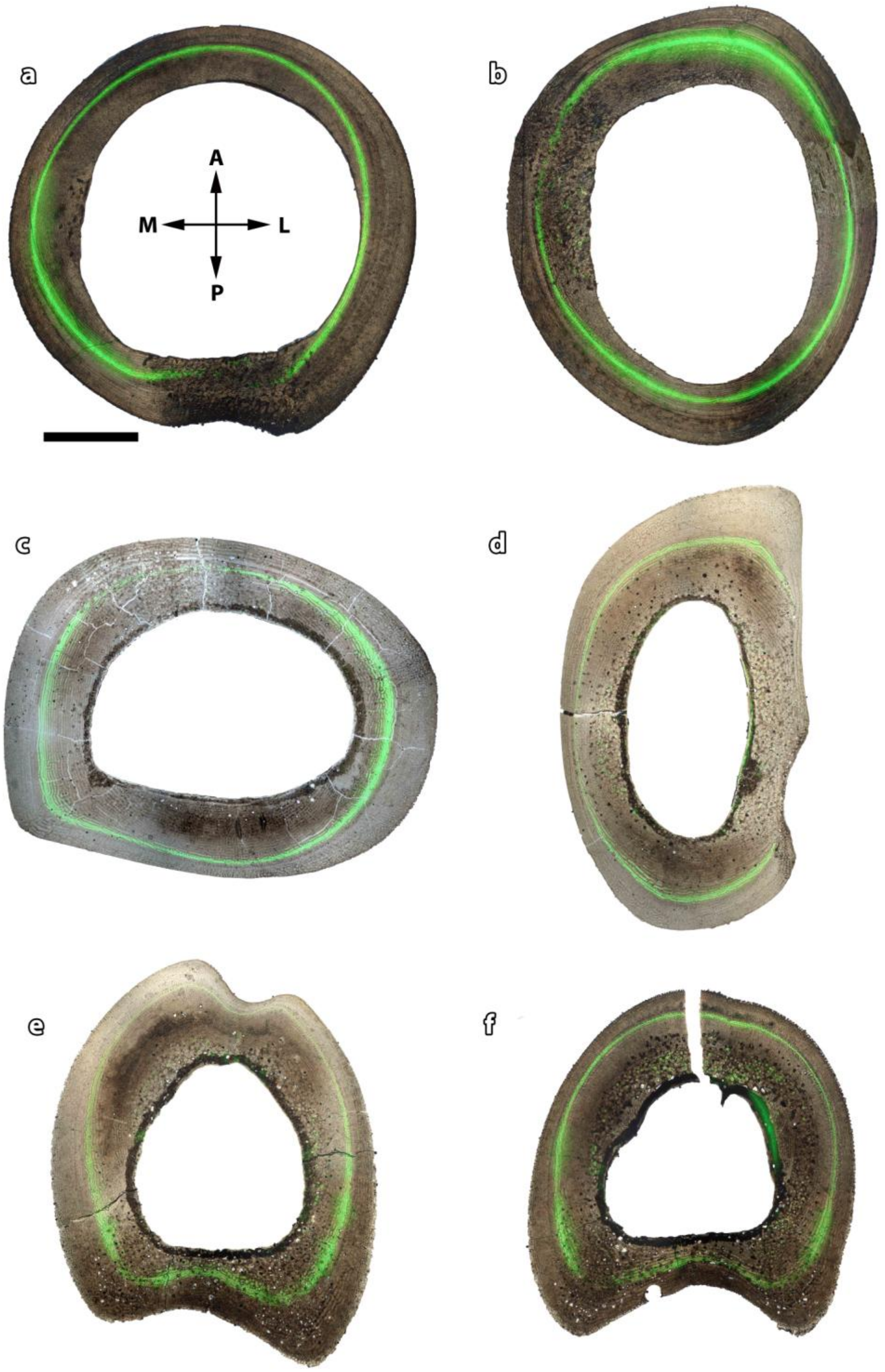

Supplementary Figure 10. Histological compositions of the hindlimb (left) and forelimb (right) bones of the 64 weeks old individual ID-23/ IPS-109290 labelled at week 13 . (a) femur. (b) humerus. (c) tibia. (d) radius. (e) metatarsus. (f) metacarpus. Scale: 5 milimetres

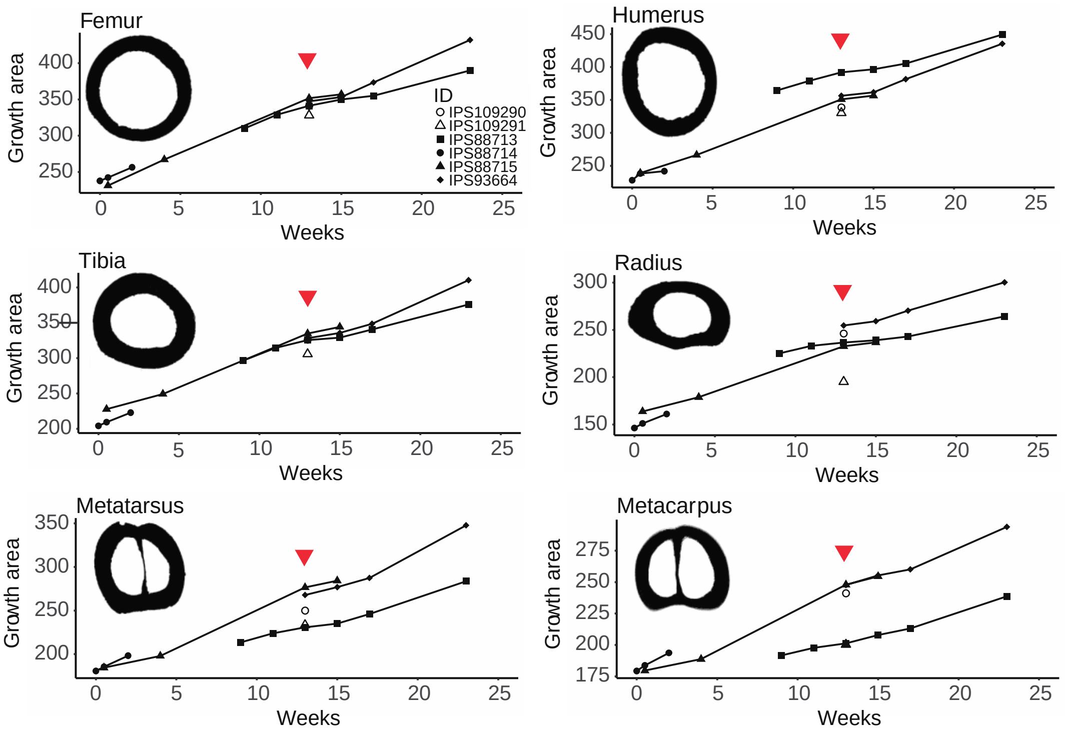

Supplementary figure 11. Amount of area between labels (in mm^2) by bone. Red arrows indicate the day of weaning (week 13 label).

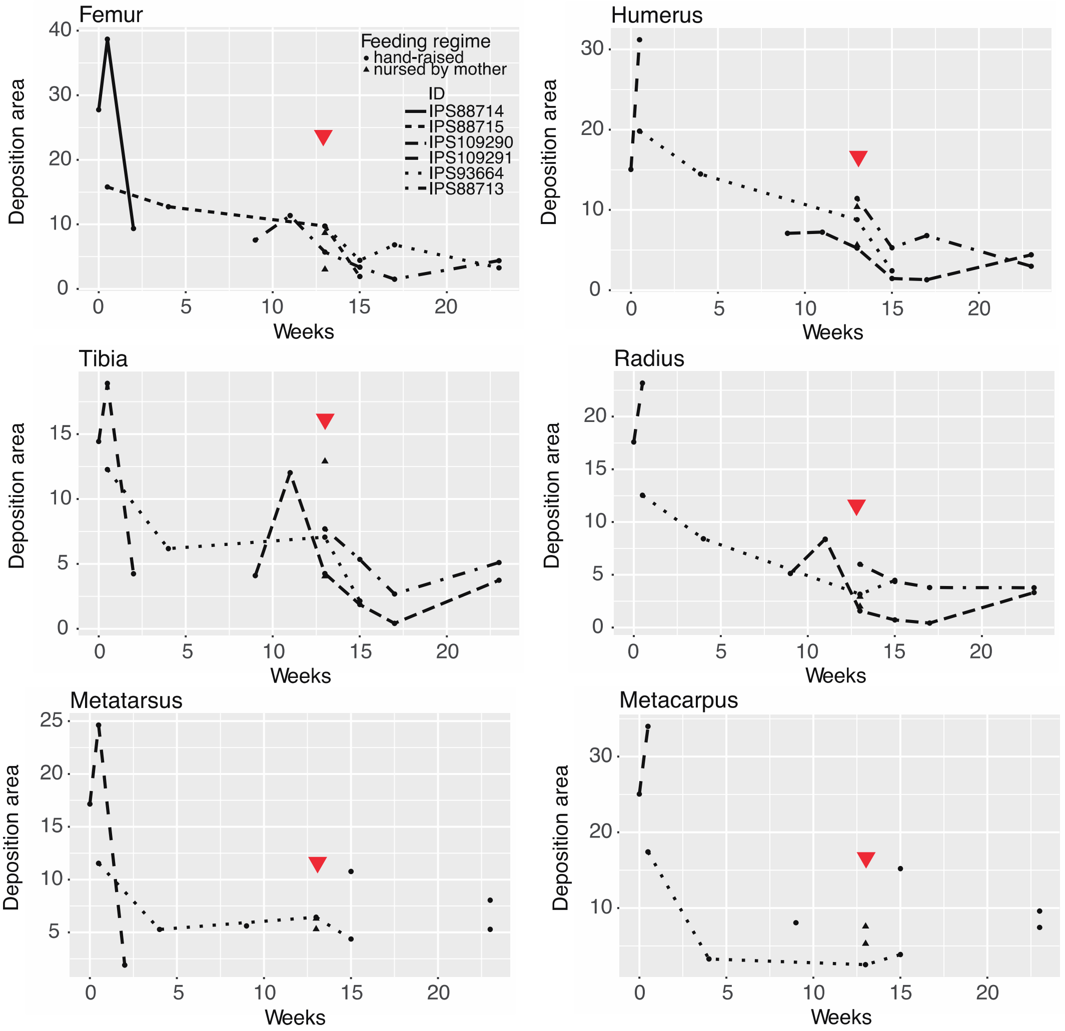

Supplementary figure 12. Labelled area (in mm^2) in the different week intervals by bone. Triangular points represent specimens nursed by their mothers (C1); circular points represent hand-raised specimens (C2). Red arrows indicate the day of weaning (week 13 label).

Supplementary Table 1. Size (in square millimetres) of tissue areas between two consecutive labels in cross-sections of various bones. Age at labelling is given in days (d) or weeks (w).

ID	regime	bone	label	full area
ID-1 / IPS-88714	hand-raised	femur	1d	237.808
ID-1 / IPS-88714	hand-raised	femur	3d	242.405
ID-1 / IPS-88714	hand-raised	femur	15d	256.541
ID-1 / IPS-88714	hand-raised	tibia	1d	204.289
ID-1 / IPS-88714	hand-raised	tibia	3d	209.419
ID-1 / IPS-88714	hand-raised	tibia	15d	223.018
ID-1 / IPS-88714	hand-raised	humerus	1d	230.360
ID-1 / IPS-88714	hand-raised	humerus	3d	236.647
ID-1 / IPS-88714	hand-raised	humerus	15d	239.420
ID-1 / IPS-88714	hand-raised	radius	1d	146.271
ID-1 / IPS-88714	hand-raised	radius	3d	151.057
ID-1 / IPS-88714	hand-raised	radius	15d	161.037
ID-1 / IPS-88714	hand-raised	metatarsus	1d	180.775
ID-1 / IPS-88714	hand-raised	metatarsus	3d	185.499
ID-1 / IPS-88714	hand-raised	metatarsus	15d	198.244
ID-1 / IPS-88714	hand-raised	metacarpus	1d	179.301
ID-1 / IPS-88714	hand-raised	metacarpus	3d	183.754
ID-1 / IPS-88714	hand-raised	metacarpus	15d	193.687
ID-2 / IPS-88715	hand-raised	femur	3d	231.333
ID-2 / IPS-88715	hand-raised	femur	30d	267.172
ID-2 / IPS-88715	hand-raised	femur	13w	351.668
ID-2 / IPS-88715	hand-raised	femur	15w	357.089
ID-2 / IPS-88715	hand-raised	tibia	3d	227.845
ID-2 / IPS-88715	hand-raised	tibia	30d	249.279
ID-2 / IPS-88715	hand-raised	tibia	13w	334.795
ID-2 / IPS-88715	hand-raised	tibia	15w	344.183
ID-2 / IPS-88715	hand-raised	humerus	3d	239.020
ID-2 / IPS-88715	hand-raised	humerus	30d	266.452
ID-2 / IPS-88715	hand-raised	humerus	13w	350.922
ID-2 / IPS-88715	hand-raised	humerus	15w	356.527
ID-2 / IPS-88715	hand-raised	radius	3d	163.746
ID-2 / IPS-88715	hand-raised	radius	30d	178.842
ID-2 / IPS-88715	hand-raised	radius	13w	232.718
ID-2 / IPS-88715	hand-raised	radius	15w	236.742
ID-2 / IPS-88715	hand-raised	metatarsus	3d	184.430
ID-2 / IPS-88715	hand-raised	metatarsus	30d	198.002
ID-2 / IPS-88715	hand-raised	metatarsus	13w	276.604
ID-2 / IPS-88715	hand-raised	metatarsus	15w	284.293
ID-2 / IPS-88715	hand-raised	metacarpus	3d	179.629
ID-2 / IPS-88715	hand-raised	metacarpus	30d	188.713

ID-2 / IPS-88715	hand-raised	metacarpus	13w	247.853
ID-2 / IPS-88715	hand-raised	metacarpus	15w	254.540
ID-3 / IPS-93664	hand-raised	femur	13w	347.394
ID-3 / IPS-93664	hand-raised	femur	15w	353.204
ID-3 / IPS-93664	hand-raised	femur	17w	373.437
ID-3 / IPS-93664	hand-raised	femur	23w	431.909
ID-3 / IPS-93664	hand-raised	tibia	13w	328.269
ID-3 / IPS-93664	hand-raised	tibia	15w	335.684
ID-3 / IPS-93664	hand-raised	tibia	17w	348.687
ID-3 / IPS-93664	hand-raised	tibia	23w	410.311
ID-3 / IPS-93664	hand-raised	humerus	13w	356.304
ID-3 / IPS-93664	hand-raised	humerus	15w	361.591
ID-3 / IPS-93664	hand-raised	humerus	17w	381.539
ID-3 / IPS-93664	hand-raised	humerus	23w	435.301
ID-3 / IPS-93664	hand-raised	radius	13w	254.631
ID-3 / IPS-93664	hand-raised	radius	15w	259.261
ID-3 / IPS-93664	hand-raised	radius	17w	270.349
ID-3 / IPS-93664	hand-raised	radius	23w	300.305
ID-3 / IPS-93664	hand-raised	metatarsus	13w	267.904
ID-3 / IPS-93664	hand-raised	metatarsus	15w	276.853
ID-3 / IPS-93664	hand-raised	metatarsus	17w	287.444
ID-3 / IPS-93664	hand-raised	metatarsus	23w	347.812
ID-3 / IPS-93664	hand-raised	metacarpus	13w	247.637
ID-3 / IPS-93664	hand-raised	metacarpus	15w	255.261
ID-3 / IPS-93664	hand-raised	metacarpus	17w	260.107
ID-3 / IPS-93664	hand-raised	metacarpus	23w	293.885
ID-4 / IPS-88713	hand-raised	femur	9w	311.594
ID-4 / IPS-88713	hand-raised	femur	11w	335.874
ID-4 / IPS-88713	hand-raised	femur	13w	345.629
ID-4 / IPS-88713	hand-raised	femur	15w	347.996
ID-4 / IPS-88713	hand-raised	femur	17w	353.429
ID-4 / IPS-88713	hand-raised	femur	23w	389.875
ID-4 / IPS-88713	hand-raised	tibia	9w	296.621
ID-4 / IPS-88713	hand-raised	tibia	11w	314.435
ID-4 / IPS-88713	hand-raised	tibia	13w	325.529
ID-4 / IPS-88713	hand-raised	tibia	15w	329.074
ID-4 / IPS-88713	hand-raised	tibia	17w	340.563
ID-4 / IPS-88713	hand-raised	tibia	23w	376.034
ID-4 / IPS-88713	hand-raised	humerus	9w	364.467
ID-4 / IPS-88713	hand-raised	humerus	11w	378.948
ID-4 / IPS-88713	hand-raised	humerus	13w	391.944
ID-4 / IPS-88713	hand-raised	humerus	15w	396.563
ID-4 / IPS-88713	hand-raised	humerus	17w	405.130
ID-4 / IPS-88713	hand-raised	humerus	23w	449.214
ID-4 / IPS-88713	hand-raised	radius	9w	225.110

ID-4 / IPS-88713	hand-raised	radius	11w	233.181
ID-4 / IPS-88713	hand-raised	radius	13w	236.701
ID-4 / IPS-88713	hand-raised	radius	15w	239.066
ID-4 / IPS-88713	hand-raised	radius	17w	243.066
ID-4 / IPS-88713	hand-raised	radius	23w	264.374
ID-4 / IPS-88713	hand-raised	metatarsus	9w	213.554
ID-4 / IPS-88713	hand-raised	metatarsus	11w	223.786
ID-4 / IPS-88713	hand-raised	metatarsus	13w	230.701
ID-4 / IPS-88713	hand-raised	metatarsus	15w	235.060
ID-4 / IPS-88713	hand-raised	metatarsus	17w	245.944
ID-4 / IPS-88713	hand-raised	metatarsus	23w	283.655
ID-4 / IPS-88713	hand-raised	metacarpus	9w	191.704
ID-4 / IPS-88713	hand-raised	metacarpus	11w	197.688
ID-4 / IPS-88713	hand-raised	metacarpus	13w	201.220
ID-4 / IPS-88713	hand-raised	metacarpus	15w	207.829
ID-4 / IPS-88713	hand-raised	metacarpus	17w	213.111
ID-4 / IPS-88713	hand-raised	metacarpus	23w	238.677
ID-23 / IPS-109290	nursed by mother	femur	13w	341.141
ID-23 / IPS-109290	nursed by mother	tibia	13w	326.214
ID-23 / IPS-109290	nursed by mother	humerus	13w	338.718
ID-23 / IPS-109290	nursed by mother	radius	13w	246.142
ID-23 / IPS-109290	nursed by mother	metatarsus	13w	249.916
ID-23 / IPS-109290	nursed by mother	metacarpus	13w	241.059
ID-24 / IPS-109291	nursed by mother	femur	13w	328.146
ID-24 / IPS-109291	nursed by mother	tibia	13w	305.770
ID-24 / IPS-109291	nursed by mother	humerus	13w	330.281
ID-24 / IPS-109291	nursed by mother	radius	13w	195.157
ID-24 / IPS-109291	nursed by mother	metatarsus	13w	233.388
ID-24 / IPS-109291	nursed by mother	metacarpus	13w	200.248

Supplementary Table 2. Growth rate (mm^2/day), canal density (%), tissue type and vascular orientation by period of each bone of the six individuals. w: week. d: days. FLC: fibro lamellar complex.

ID	regime	bone	period	growth rate	canal density
ID-1 / IPS-88714	hand-raised	femur	prenatal	NA	10.992
ID-1 / IPS-88714	hand-raised	femur	1d-3d	1.532	NA
ID-1 / IPS-88714	hand-raised	femur	3d-15d	1.087	NA
ID-1 / IPS-88714	hand-raised	tibia	prenatal	NA	13.137
ID-1 / IPS-88714	hand-raised	tibia	1d-3d	1.71	NA
ID-1 / IPS-88714	hand-raised	tibia	3d-15d	1.046	NA
ID-1 / IPS-88714	hand-raised	humerus	prenatal	NA	17.158
ID-1 / IPS-88714	hand-raised	humerus	1d-3d	2.096	NA
ID-1 / IPS-88714	hand-raised	humerus	3d-15d	0.768	NA
ID-1 / IPS-88714	hand-raised	radius	prenatal	NA	12.601
ID-1 / IPS-88714	hand-raised	radius	1d-3d	1.595	NA
ID-1 / IPS-88714	hand-raised	radius	3d-15d	0.981	NA
ID-1 / IPS-88714	hand-raised	metatarsus	prenatal	NA	17.158
ID-1 / IPS-88714	hand-raised	metatarsus	1d-3d	1.575	NA
ID-1 / IPS-88714	hand-raised	metatarsus	3d-15d	0.28	NA
ID-1 / IPS-88714	hand-raised	metacarpus	prenatal	NA	9.383
ID-1 / IPS-88714	hand-raised	metacarpus	1d-3d	1.484	NA
ID-1 / IPS-88714	hand-raised	metacarpus	3d-15d	0.764	NA
ID-2 / IPS-88715	hand-raised	femur	prenatal	NA	14.745
ID-2 / IPS-88715	hand-raised	femur	3d-30d	1.327	8.311
ID-2 / IPS-88715	hand-raised	femur	30d-13w	1.457	8.300
ID-2 / IPS-88715	hand-raised	femur	13w-15w	0.387	NA
ID-2 / IPS-88715	hand-raised	tibia	prenatal	NA	11.260
ID-2 / IPS-88715	hand-	tibia	3d-30d	0.794	12.601

	raised				
ID-2 / IPS-88715	hand-raised	tibia	30d-13w	1.474	11.000
ID-2 / IPS-88715	hand-raised	tibia	13w-15w	0.67	NA
ID-2 / IPS-88715	hand-raised	humerus	prenatal	NA	NA
ID-2 / IPS-88715	hand-raised	humerus	3d-30d	1.016	15.818
ID-2 / IPS-88715	hand-raised	humerus	30d-13w	1.456	18.499
ID-2 / IPS-88715	hand-raised	humerus	13w-15w	0.400	NA
ID-2 / IPS-88715	hand-raised	radius	prenatal	NA	16.086
ID-2 / IPS-88715	hand-raised	radius	3d-30d	0.559	11.796
ID-2 / IPS-88715	hand-raised	radius	30d-13w	0.929	14.700
ID-2 / IPS-88715	hand-raised	radius	13w-15w	0.287	NA
ID-2 / IPS-88715	hand-raised	metatarsus	prenatal	NA	13.673
ID-2 / IPS-88715	hand-raised	metatarsus	3d-30d	0.503	NA
ID-2 / IPS-88715	hand-raised	metatarsus	30d-13w	1.355	9.900
ID-2 / IPS-88715	hand-raised	metatarsus	13w-15w	0.549	NA
ID-2 / IPS-88715	hand-raised	metacarpus	prenatal	NA	13.405
ID-2 / IPS-88715	hand-raised	metacarpus	3d-30d	0.336	NA
ID-2 / IPS-88715	hand-raised	metacarpus	30d-13w	1.02	22.300
ID-2 / IPS-88715	hand-raised	metacarpus	13w-15w	0.477	NA
ID-3 / IPS-93664	hand-raised	femur	before 13w	NA	11.800
ID-3 / IPS-93664	hand-raised	femur	13w-15w	0.415	NA
ID-3 / IPS-93664	hand-raised	femur	15w-17w	1.445	7.500
ID-3 / IPS-93664	hand-raised	femur	17w-23w	1.271	
ID-3 / IPS-93664	hand-raised	tibia	before 13w	NA	13.400
ID-3 / IPS-93664	hand-raised	tibia	13w-15w	0.53	NA
ID-3 / IPS-93664	hand-raised	tibia	15w-17w	0.929	7.200
ID-3 / IPS-93664	hand-raised	tibia	17w-23w	1.34	

ID-3 / IPS-93664	hand-raised	humerus	before 13w	NA	18.500
ID-3 / IPS-93664	hand-raised	humerus	13w-15w	0.378	NA
ID-3 / IPS-93664	hand-raised	humerus	15w-17w	1.425	12.600
ID-3 / IPS-93664	hand-raised	humerus	17w-23w	1.169	
ID-3 / IPS-93664	hand-raised	radius	prenatal	NA	NA
ID-3 / IPS-93664	hand-raised	radius	before 13w	NA	18.800
ID-3 / IPS-93664	hand-raised	radius	13w-15w	0.331	NA
ID-3 / IPS-93664	hand-raised	radius	15w-17w	0.792	11.300
ID-3 / IPS-93664	hand-raised	radius	17w-23w	0.651	
ID-3 / IPS-93664	hand-raised	metatarsus	prenatal	NA	NA
ID-3 / IPS-93664	hand-raised	metatarsus	before 13w	NA	11.800
ID-3 / IPS-93664	hand-raised	metatarsus	13w-15w	0.639	NA
ID-3 / IPS-93664	hand-raised	metatarsus	15w-17w	0.757	6.700
ID-3 / IPS-93664	hand-raised	metatarsus	17w-23w	1.312	
ID-3 / IPS-93664	hand-raised	metacarpus	prenatal	NA	NA
ID-3 / IPS-93664	hand-raised	metacarpus	before 13w	NA	13.100
ID-3 / IPS-93664	hand-raised	metacarpus	13w-15w	0.544	NA
ID-3 / IPS-93664	hand-raised	metacarpus	15w-17w	0.346	12.900
ID-3 / IPS-93664	hand-raised	metacarpus	17w-23w	0.734	
ID-4 / IPS-88713	hand-raised	femur	before 9w	NA	NA
ID-4 / IPS-88713	hand-raised	femur	9w-11w	NA	10.700
ID-4 / IPS-88713	hand-raised	femur	11w-13w	NA	
ID-4 / IPS-88713	hand-raised	femur	13w-15w	NA	NA
ID-4 / IPS-88713	hand-raised	femur	15w-17w	0.368	7.200
ID-4 / IPS-88713	hand-raised	femur	17w-23w	0.831	
ID-4 / IPS-88713	hand-raised	tibia	before 9w	NA	0.000
ID-4 / IPS-88713	hand-raised	tibia	9w-11w	NA	14.500

ID-4 / IPS-88713	hand-raised	tibia	11w-13w	NA	
ID-4 / IPS-88713	hand-raised	tibia	13w-15w	NA	NA
ID-4 / IPS-88713	hand-raised	tibia	15w-17w	0.821	9.900
ID-4 / IPS-88713	hand-raised	tibia	17w-23w	0.845	
ID-4 / IPS-88713	hand-raised	humerus	before 9w	NA	NA
ID-4 / IPS-88713	hand-raised	humerus	9w-11w	NA	13.100
ID-4 / IPS-88713	hand-raised	humerus	11w-13w	NA	
ID-4 / IPS-88713	hand-raised	humerus	13w-15w	NA	NA
ID-4 / IPS-88713	hand-raised	humerus	15w-17w	0.612	11.000
ID-4 / IPS-88713	hand-raised	humerus	17w-23w	1.050	
ID-4 / IPS-88713	hand-raised	radius	before 9w	NA	NA
ID-4 / IPS-88713	hand-raised	radius	9w-11w	NA	20.400
ID-4 / IPS-88713	hand-raised	radius	11w-13w	NA	
ID-4 / IPS-88713	hand-raised	radius	13w-15w	NA	NA
ID-4 / IPS-88713	hand-raised	radius	15w-17w	0.286	11.800
ID-4 / IPS-88713	hand-raised	radius	17w-23w	0.507	
ID-4 / IPS-88713	hand-raised	metatarsus	prenatal	NA	NA
ID-4 / IPS-88713	hand-raised	metatarsus	before 9w	NA	NA
ID-4 / IPS-88713	hand-raised	metatarsus	9w-11w	NA	24.100
ID-4 / IPS-88713	hand-raised	metatarsus	11w-13w	NA	
ID-4 / IPS-88713	hand-raised	metatarsus	13w-15w	NA	NA
ID-4 / IPS-88713	hand-raised	metatarsus	15w-17w	0.777	11.800
ID-4 / IPS-88713	hand-raised	metatarsus	17w-23w	0.898	
ID-4 / IPS-88713	hand-raised	metacarpus	before 9w	NA	0.000
ID-4 / IPS-88713	hand-raised	metacarpus	9w-11w	NA	8.300
ID-4 / IPS-88713	hand-raised	metacarpus	11w-13w	NA	
ID-4 / IPS-88713	hand-raised	metacarpus	13w-15w	NA	NA

ID-4 / IPS-88713	hand-raised	metacarpus	15w-17w	0.377	7.500
ID-4 / IPS-88713	hand-raised	metacarpus	17w-23w	0.609	
ID-23 / IPS-109290	nursed by mother	femur	before 13w	NA	13.100
ID-23 / IPS-109290	nursed by mother	femur	after 13w	NA	10.500
ID-23 / IPS-109290	nursed by mother	tibia	before 13w	NA	9.400
ID-23 / IPS-109290	nursed by mother	tibia	after 13w	NA	8.000
ID-23 / IPS-109290	nursed by mother	humerus	before 13w	NA	18.500
ID-23 / IPS-109290	nursed by mother	humerus	after 13w	NA	13.700
ID-23 / IPS-109290	nursed by mother	radius	before 13w	NA	14.200
ID-23 / IPS-109290	nursed by mother	radius	after 13w	NA	12.600
ID-23 / IPS-109290	nursed by mother	metatarsus	before 13w	NA	5.900
ID-23 / IPS-109290	nursed by mother	metatarsus	after 13w	NA	13.400
ID-23 / IPS-109290	nursed by mother	metacarpus	before 13w	NA	8.300
ID-23 / IPS-109290	nursed by mother	metacarpus	after 13w	NA	4.800
ID-24 / IPS-109291	nursed by mother	femur	before 13w	1.318	12.900
ID-24 / IPS-109291	nursed by mother	femur	after 13w	0.446	6.200
ID-24 / IPS-109291	nursed by mother	tibia	before 13w	1.736	7.000
ID-24 / IPS-109291	nursed by mother	tibia	after 13w	0.326	11.500
ID-24 / IPS-109291	nursed by mother	humerus	before 13w	0.850	8.600
ID-24 / IPS-109291	nursed by mother	humerus	after 13w	0.279	11.300
ID-24 / IPS-109291	nursed by mother	radius	before 13w	1.100	8.800
ID-24 / IPS-109291	nursed by mother	radius	after 13w	0.267	8.600
ID-24 / IPS-109291	nursed by mother	metatarsus	before 13w	1.264	7.500
ID-24 / IPS-109291	nursed by mother	metatarsus	after 13w	0.433	9.100
ID-24 / IPS-109291	nursed by mother	metacarpus	before 13w	0.838	9.700
ID-24 / IPS-109291	nursed by mother	metacarpus	after 13w	0.151	8.000

Supplmentary Table 3. Feed regime information based on the quantity of milk intake (i.e. artificial milk) and vegetal pellets (in grams per millilitre) together to the daily body mass (in kilograms) for the hand-raised individuals (C2 group).

ID	Day	Milk intake (ml)	Vegetal pellets (g/ml)	Bodymass (kg)
ID-1 / IPS-88714	1	-	0	11.5
ID-1 / IPS-88714	2	-	0	-
ID-1 / IPS-88714	3	1050	0	-
ID-1 / IPS-88714	4	1300	0	13.3
ID-1 / IPS-88714	5	900	0	14.0
ID-1 / IPS-88714	6	900	0	14.7
ID-1 / IPS-88714	7	1100	0	14.8
ID-1 / IPS-88714	8	1200	0	15.4
ID-1 / IPS-88714	9	1200	0	16.0
ID-1 / IPS-88714	10	1200	0	16.7
ID-1 / IPS-88714	11	1150	0	18.1
ID-1 / IPS-88714	12	1150	0	18.0
ID-1 / IPS-88714	13	1200	0	17.7
ID-1 / IPS-88714	14	800	0	18.0
ID-1 / IPS-88714	15	800	0	18.2
ID-1 / IPS-88714	16	1200	0	18.9
ID-1 / IPS-88714	17	1800	0	19.5
ID-2 / IPS-88715	1	-	0	-
ID-2 / IPS-88715	2	-	0	-
ID-2 / IPS-88715	3	850	0	10.5
ID-2 / IPS-88715	4	1000	0	11.1
ID-2 / IPS-88715	5	950	0	11.7
ID-2 / IPS-88715	6	1050	0	12.3
ID-2 / IPS-88715	7	1050	0	12.6
ID-2 / IPS-88715	8	1050	0	13.0
ID-2 / IPS-88715	9	1150	0	12.8
ID-2 / IPS-88715	10	900	0	13.4
ID-2 / IPS-88715	11	900	0	13.3
ID-2 / IPS-88715	12	900	0	13.9
ID-2 / IPS-88715	13	1100	0	13.9
ID-2 / IPS-88715	14	1200	0	14.4
ID-2 / IPS-88715	15	800	0	14.8
ID-2 / IPS-88715	16	1200	0	15.0
ID-2 / IPS-88715	17	1200	0	15.3
ID-2 / IPS-88715	18	1200	0	15.5
ID-2 / IPS-88715	19	800	0	16.1
ID-2 / IPS-88715	20	1200	0	16.0
ID-2 / IPS-88715	21	1200	0	16.4
ID-2 / IPS-88715	22	1200	0	17.1

ID-2 / IPS-88715	23	1200	0	17.4
ID-2 / IPS-88715	24	900	0	17.4
ID-2 / IPS-88715	25	700	0	17.9
ID-2 / IPS-88715	26	700	120	18.0
ID-2 / IPS-88715	27	700	-	18.7
ID-2 / IPS-88715	28	800	-	18.9
ID-2 / IPS-88715	29	800	100	18.9
ID-2 / IPS-88715	30	800	100	19.4
ID-2 / IPS-88715	31	1200	-	19.7
ID-2 / IPS-88715	32	500	100	19.3
ID-2 / IPS-88715	33	1100	100	19.7
ID-2 / IPS-88715	34	1000	100	20.2
ID-2 / IPS-88715	35	1000	140	20.6
ID-2 / IPS-88715	36	1200	140	20.8
ID-2 / IPS-88715	37	1200	140	21.2
ID-2 / IPS-88715	38	1200	140	21.4
ID-2 / IPS-88715	39	1200	160	21.4
ID-2 / IPS-88715	40	1200	160	21.8
ID-2 / IPS-88715	41	1200	160	22.0
ID-2 / IPS-88715	42	1400	160	22.8
ID-2 / IPS-88715	43	1400	160	23.0
ID-2 / IPS-88715	44	1400	160	23.4
ID-2 / IPS-88715	45	1400	160	23.6
ID-2 / IPS-88715	46	1400	160	23.6
ID-2 / IPS-88715	47	1400	200	24.2
ID-2 / IPS-88715	48	1400	200	24.8
ID-2 / IPS-88715	49	1400	200	25.2
ID-2 / IPS-88715	50	1400	200	25.4
ID-2 / IPS-88715	51	1400	200	25.8
ID-2 / IPS-88715	52	1400	220	26.8
ID-2 / IPS-88715	53	1400	240	26.6
ID-2 / IPS-88715	54	1400	240	27.4
ID-2 / IPS-88715	55	1400	240	28.0
ID-2 / IPS-88715	56	1400	260	28.6
ID-2 / IPS-88715	57	1400	300	28.6
ID-2 / IPS-88715	58	1400	300	29.0
ID-2 / IPS-88715	59	1400	300	29.0
ID-2 / IPS-88715	60	1400	300	29.6
ID-2 / IPS-88715	61	1400	300	29.6
ID-2 / IPS-88715	62	1400	300	29.6
ID-2 / IPS-88715	63	1400	300	30.0
ID-2 / IPS-88715	64	1400	340	31.0
ID-2 / IPS-88715	65	1400	400	31.2
ID-2 / IPS-88715	66	1400	400	31.6
ID-2 / IPS-88715	67	1400	400	31.6

ID-2 / IPS-88715	68	1400	400	32.0
ID-2 / IPS-88715	69	1400	400	32.2
ID-2 / IPS-88715	70	1400	400	33.0
ID-2 / IPS-88715	71	1400	400	33.2
ID-2 / IPS-88715	72	1400	400	33.0
ID-2 / IPS-88715	73	1400	400	33.8
ID-2 / IPS-88715	74	1400	400	34.0
ID-2 / IPS-88715	75	1400	400	34.0
ID-2 / IPS-88715	76	1400	400	35.0
ID-2 / IPS-88715	77	1400	400	35.2
ID-2 / IPS-88715	78	1400	400	35.4
ID-2 / IPS-88715	79	1400	400	35.6
ID-2 / IPS-88715	80	1400	400	35.6
ID-2 / IPS-88715	81	1400	400	35.6
ID-2 / IPS-88715	82	1400	400	36.4
ID-2 / IPS-88715	83	1400	400	36.6
ID-2 / IPS-88715	84	700	400	37.0
ID-2 / IPS-88715	85	1400	400	37.2
ID-2 / IPS-88715	86	1400	400	-
ID-2 / IPS-88715	87	1400	400	-
ID-2 / IPS-88715	88	1400	400	38.0
ID-2 / IPS-88715	89	0	400	38.0
ID-2 / IPS-88715	90	0	400	-
ID-2 / IPS-88715	91	0	350	-
ID-2 / IPS-88715	92	0	150	-
ID-2 / IPS-88715	93	0	150	-
ID-2 / IPS-88715	94	0	150	-
ID-2 / IPS-88715	95	0	150	-
ID-2 / IPS-88715	96	0	150	-
ID-2 / IPS-88715	97	0	150	-
ID-2 / IPS-88715	98	0	100	-
ID-2 / IPS-88715	99	0	100	-
ID-2 / IPS-88715	100	0	100	-
ID-2 / IPS-88715	101	0	-	-
ID-2 / IPS-88715	102	0	-	-
ID-2 / IPS-88715	103	0	-	-
ID-2 / IPS-88715	104	0	-	-
ID-2 / IPS-88715	105	0	-	42.0
ID-3 / IPS-93664	1	-	0	-
ID-3 / IPS-93664	2	-	0	-
ID-3 / IPS-93664	3	850	0	10.5
ID-3 / IPS-93664	4	1000	0	10.7
ID-3 / IPS-93664	5	1000	0	11.0
ID-3 / IPS-93664	6	1050	0	11.3
ID-3 / IPS-93664	7	1050	0	11.7

ID-3 / IPS-93664	8	1100	0	12.6
ID-3 / IPS-93664	9	1150	0	13.0
ID-3 / IPS-93664	10	900	0	13.2
ID-3 / IPS-93664	11	900	0	13.0
ID-3 / IPS-93664	12	900	0	13.2
ID-3 / IPS-93664	13	1100	0	13.6
ID-3 / IPS-93664	14	1200	0	13.9
ID-3 / IPS-93664	15	800	0	14.7
ID-3 / IPS-93664	16	1200	0	14.7
ID-3 / IPS-93664	17	1200	0	15.3
ID-3 / IPS-93664	18	1200	0	15.6
ID-3 / IPS-93664	19	1200	0	15.9
ID-3 / IPS-93664	20	1200	0	16.4
ID-3 / IPS-93664	21	1200	0	16.7
ID-3 / IPS-93664	22	1200	0	17.3
ID-3 / IPS-93664	23	1200	0	17.4
ID-3 / IPS-93664	24	1200	0	17.8
ID-3 / IPS-93664	25	1100	0	18.0
ID-3 / IPS-93664	26	800	200	18.5
ID-3 / IPS-93664	27	700	60	18.5
ID-3 / IPS-93664	28	800	85	18.5
ID-3 / IPS-93664	29	800	100	19.1
ID-3 / IPS-93664	30	800	100	19.0
ID-3 / IPS-93664	31	1800	-	19.5
ID-3 / IPS-93664	32	1800	-	19.5
ID-3 / IPS-93664	33	1800	100	20.0
ID-3 / IPS-93664	34	1200	100	20.2
ID-3 / IPS-93664	35	1000	140	20.8
ID-3 / IPS-93664	36	1200	140	20.8
ID-3 / IPS-93664	37	1200	140	21.4
ID-3 / IPS-93664	38	1200	140	21.4
ID-3 / IPS-93664	39	1200	160	21.6
ID-3 / IPS-93664	40	1200	160	21.8
ID-3 / IPS-93664	41	1200	160	22.4
ID-3 / IPS-93664	42	1400	160	22.6
ID-3 / IPS-93664	43	1400	160	23.0
ID-3 / IPS-93664	44	1400	160	23.6
ID-3 / IPS-93664	45	1400	160	23.4
ID-3 / IPS-93664	46	1400	160	23.6
ID-3 / IPS-93664	47	1400	200	24.4
ID-3 / IPS-93664	48	1400	200	24.6
ID-3 / IPS-93664	49	1400	200	25.2
ID-3 / IPS-93664	50	1400	200	25.2
ID-3 / IPS-93664	51	1400	200	25.6
ID-3 / IPS-93664	52	1400	200	26.2

ID-3 / IPS-93664	53	1400	240	26.4
ID-3 / IPS-93664	54	1400	240	27.2
ID-3 / IPS-93664	55	1400	240	27.6
ID-3 / IPS-93664	56	1400	260	28.2
ID-3 / IPS-93664	57	1400	300	28.2
ID-3 / IPS-93664	58	1300	280	27.8
ID-3 / IPS-93664	59	1400	260	28.6
ID-3 / IPS-93664	60	1400	260	29.2
ID-3 / IPS-93664	61	1400	260	29.8
ID-3 / IPS-93664	62	1400	260	30.0
ID-3 / IPS-93664	63	1400	260	30.6
ID-3 / IPS-93664	64	1400	280	31.0
ID-3 / IPS-93664	65	1400	300	31.6
ID-3 / IPS-93664	66	1400	300	31.6
ID-3 / IPS-93664	67	1400	300	31.8
ID-3 / IPS-93664	68	1400	300	32.2
ID-3 / IPS-93664	69	1400	300	33.0
ID-3 / IPS-93664	70	1400	300	33.2
ID-3 / IPS-93664	71	1400	300	33.8
ID-3 / IPS-93664	72	1400	300	33.6
ID-3 / IPS-93664	73	1400	300	33.8
ID-3 / IPS-93664	74	1400	300	34.2
ID-3 / IPS-93664	75	1400	300	34.2
ID-3 / IPS-93664	76	1400	300	35.0
ID-3 / IPS-93664	77	1400	300	35.4
ID-3 / IPS-93664	78	1400	300	35.8
ID-3 / IPS-93664	79	1400	300	36.5
ID-3 / IPS-93664	80	1400	300	37.6
ID-3 / IPS-93664	81	1400	300	37.4
ID-3 / IPS-93664	82	1400	300	38.2
ID-3 / IPS-93664	83	1400	300	38.6
ID-3 / IPS-93664	84	700	300	38.8
ID-3 / IPS-93664	85	1400	340	39.2
ID-3 / IPS-93664	86	1400	400	-
ID-3 / IPS-93664	87	1400	400	-
ID-3 / IPS-93664	88	1400	400	-
ID-3 / IPS-93664	89	0	400	41.0
ID-3 / IPS-93664	90	0	400	-
ID-3 / IPS-93664	91	0	350	-
ID-3 / IPS-93664	92	0	150	-
ID-3 / IPS-93664	93	0	150	-
ID-3 / IPS-93664	94	0	150	-
ID-3 / IPS-93664	95	0	150	-
ID-3 / IPS-93664	96	0	150	-
ID-3 / IPS-93664	97	0	150	-

ID-3 / IPS-93664	98	0	100	-
ID-3 / IPS-93664	99	0	100	-
ID-3 / IPS-93664	100	0	100	-
ID-3 / IPS-93664	101	0	-	44.0
ID-3 / IPS-93664	102-114	0	-	-
ID-3 / IPS-93664	115	0	-	52
ID-3 / IPS-93664	116-160	0	-	-
ID-3 / IPS-93664	161	0	-	70
ID-3 / IPS-93664	161-203	0	-	-
ID-3 / IPS-93664	204	0	-	72
ID-3 / IPS-93664	205-240	0	-	-
ID-3 / IPS-93664	241	0	-	75
ID-3 / IPS-93664	242-289	0	-	-
ID-3 / IPS-93664	290	0	-	80
ID-3 / IPS-93664	291-328	0	-	-
ID-3 / IPS-93664	329	0	-	92.5
ID-3 / IPS-93664	330-331	0	-	-
ID-3 / IPS-93664	332	0	-	-
ID-4 / IPS-88713	1	-	0	10.0
ID-4 / IPS-88713	2	-	0	-
ID-4 / IPS-88713	3	1150	0	-
ID-4 / IPS-88713	4	1100	0	-
ID-4 / IPS-88713	5	1000	0	-
ID-4 / IPS-88713	6	1000	0	11.2
ID-4 / IPS-88713	7	550	0	11.3
ID-4 / IPS-88713	8	600	0	11.5
ID-4 / IPS-88713	9	1150	0	11.6
ID-4 / IPS-88713	10	1200	0	11.7
ID-4 / IPS-88713	11	550	0	12.4
ID-4 / IPS-88713	12	1000	0	12.0
ID-4 / IPS-88713	13	850	0	12.8
ID-4 / IPS-88713	14	600	0	13.1
ID-4 / IPS-88713	15	600	0	13.7
ID-4 / IPS-88713	16	850	0	13.8
ID-4 / IPS-88713	17	900	0	14.1
ID-4 / IPS-88713	18	900	0	14.2
ID-4 / IPS-88713	19	900	0	14.5
ID-4 / IPS-88713	20	900	0	14.7
ID-4 / IPS-88713	21	700	0	15.2
ID-4 / IPS-88713	22	1200	0	15.4
ID-4 / IPS-88713	23	1050	0	16.2
ID-4 / IPS-88713	24	1100	0	16.3
ID-4 / IPS-88713	25	1050	0	16.9
ID-4 / IPS-88713	26	1200	0	16.7
ID-4 / IPS-88713	27	1200	0	-

ID-4 / IPS-88713	28	1200	0	17.5
ID-4 / IPS-88713	29	1250	0	18.0
ID-4 / IPS-88713	30	1350	0	18.5
ID-4 / IPS-88713	31	1350	0	19.4
ID-4 / IPS-88713	32	950	0	19.7
ID-4 / IPS-88713	33	1000	0	19.8
ID-4 / IPS-88713	34	1000	0	20.0
ID-4 / IPS-88713	35	1000	0	21.0
ID-4 / IPS-88713	36	1200	0	21.4
ID-4 / IPS-88713	37	1100	0	21.6
ID-4 / IPS-88713	38	950	0	21.9
ID-4 / IPS-88713	39	1000	0	22.5
ID-4 / IPS-88713	40	1000	0	22.8
ID-4 / IPS-88713	41	1000	0	23.2
ID-4 / IPS-88713	42	1000	0	23.7
ID-4 / IPS-88713	43	1200	0	23.8
ID-4 / IPS-88713	44	1100	0	24.8
ID-4 / IPS-88713	45	1100	0	25.0
ID-4 / IPS-88713	46	1100	0	25.4
ID-4 / IPS-88713	47	1100	0	26.0
ID-4 / IPS-88713	48	1000	0	26.0
ID-4 / IPS-88713	49	1000	0	26.4
ID-4 / IPS-88713	50	1000	0	26.8
ID-4 / IPS-88713	51	1000	0	27.0
ID-4 / IPS-88713	52	1000	0	27.4
ID-4 / IPS-88713	53	1000	0	28.2
ID-4 / IPS-88713	54	1000	0	28.6
ID-4 / IPS-88713	55	1100	0	28.8
ID-4 / IPS-88713	56	1200	0	29.6
ID-4 / IPS-88713	57	1200	0	30.2
ID-4 / IPS-88713	58	1200	0	30.4
ID-4 / IPS-88713	59	1200	0	31.2
ID-4 / IPS-88713	60	1200	0	31.4
ID-4 / IPS-88713	61	1200	0	31.6
ID-4 / IPS-88713	62	1200	0	32.4
ID-4 / IPS-88713	63	1200	0	33.0
ID-4 / IPS-88713	64	1200	0	33.4
ID-4 / IPS-88713	65	1200	0	33.4
ID-4 / IPS-88713	66	1200	0	33.8
ID-4 / IPS-88713	67	1200	0	-
ID-4 / IPS-88713	68	1200	0	34.2
ID-4 / IPS-88713	69	1200	0	34.6
ID-4 / IPS-88713	70	1200	0	35.0
ID-4 / IPS-88713	71	1200	0	35.4
ID-4 / IPS-88713	72	1200	0	35.8

ID-4 / IPS-88713	73	1200	0	36.4
ID-4 / IPS-88713	74	600	0	36.8
ID-4 / IPS-88713	75	1200	0	34.0
ID-4 / IPS-88713	76	1200	0	37.2
ID-4 / IPS-88713	77	1200	0	37.8
ID-4 / IPS-88713	78	1200	0	38.0
ID-4 / IPS-88713	79	600	0	38.2
ID-4 / IPS-88713	80	1200	0	38.2
ID-4 / IPS-88713	81	1200	0	-
ID-4 / IPS-88713	82	1200	0	38.6
ID-4 / IPS-88713	83	1200	0	39.2
ID-4 / IPS-88713	84	1200	0	39.4
ID-4 / IPS-88713	85	600	0	40.0
ID-4 / IPS-88713	86	1200	0	39.2
ID-4 / IPS-88713	87	1200	0	40.4
ID-4 / IPS-88713	88	1200	200	40.2
ID-4 / IPS-88713	89	1200	20	41.0
ID-4 / IPS-88713	90	1200	200	41.6
ID-4 / IPS-88713	91	1200	200	41.6
ID-4 / IPS-88713	92	1200	200	42.4
ID-4 / IPS-88713	93	1200	200	42.4
ID-4 / IPS-88713	94	1000	300	42.6
ID-4 / IPS-88713	95	0	300	-
ID-4 / IPS-88713	96	0	300	42.6
ID-4 / IPS-88713	97	0	300	42.6
ID-4 / IPS-88713	98	0	300	42.6
ID-4 / IPS-88713	99	0	300	43.0
ID-4 / IPS-88713	100	0	300	42.8
ID-4 / IPS-88713	101	0	300	43.0
ID-4 / IPS-88713	102	0	300	42.2
ID-4 / IPS-88713	103	0	300	42.4
ID-4 / IPS-88713	104	0	300	42.2
ID-4 / IPS-88713	105	0	150	42.4
ID-4 / IPS-88713	106	0	150	42.4
ID-4 / IPS-88713	107	0	150	42.8
ID-4 / IPS-88713	108	0	150	43.0
ID-4 / IPS-88713	109	0	150	43.2
ID-4 / IPS-88713	110	0	150	-
ID-4 / IPS-88713	111	0	150	42.6
ID-4 / IPS-88713	112	0	150	42.2
ID-4 / IPS-88713	113	0	150	42.4
ID-4 / IPS-88713	114	0	150	42.8
ID-4 / IPS-88713	115	0	150	43.8
ID-4 / IPS-88713	116	0	150	45.0
ID-4 / IPS-88713	117	0	150	44.0

ID-4 / IPS-88713	118	0	100	44.0
ID-4 / IPS-88713	119	0	100	44.8
ID-4 / IPS-88713	120	0	100	44.8
ID-4 / IPS-88713	121	0	100	45.0
ID-4 / IPS-88713	122	0	100	46.0
ID-4 / IPS-88713	123	0	100	46.4
ID-4 / IPS-88713	124	0	100	-
ID-4 / IPS-88713	125	0	100	46.8
ID-4 / IPS-88713	126	0	100	47.2
ID-4 / IPS-88713	127	0	100	47.8
ID-4 / IPS-88713	128	0	100	48.0
ID-4 / IPS-88713	129	0	100	47.8
ID-4 / IPS-88713	130	0	100	48.4
ID-4 / IPS-88713	131	0	100	48.8
ID-4 / IPS-88713	132	0	100	49.0
ID-4 / IPS-88713	133	0	100	49.4
ID-4 / IPS-88713	134	0	100	50.6
ID-4 / IPS-88713	135	0	100	50.5
ID-4 / IPS-88713	136	0	50	50.5
ID-4 / IPS-88713	137	0	50	51.5
ID-4 / IPS-88713	138	0	50	51.5
ID-4 / IPS-88713	139	0	50	51.0
ID-4 / IPS-88713	140	0	50	-
ID-4 / IPS-88713	141-164	0	-	-
ID-4 / IPS-88713	165	0	-	58.5
ID-4 / IPS-88713	166-206	0	-	-
ID-4 / IPS-88713	207	0	-	64
ID-4 / IPS-88713	208-248	0	-	-
ID-4 / IPS-88713	249	0	-	72.5
ID-4 / IPS-88713	250-290	0	-	-
ID-4 / IPS-88713	291	0	-	83
ID-4 / IPS-88713	292-330	0	-	-
ID-4 / IPS-88713	331	0	-	92
ID-4 / IPS-88713	332	0	-	-
ID-4 / IPS-88713	333	0	-	92

Supplementary Table 4. Standardization of the fluorochrome-stained (labelled) areas (in square millimetres) by applying the correction factor based on the common label (i.e. 13weeks) on the measured labelled areas (also in square millimetres). hand: hand-raised regime; nursed: individuals that remained with their mothers.

ID	regime	bone	period	measured area	correction factor	standarized area
ID-1 / IPS-88714	hand	femur	Birth	19.258	1.441	27.760
ID-1 / IPS-88714	hand	femur	3 days	27.356	1.414	38.685
ID-1 / IPS-88714	hand	femur	15 days	7.003	1.336	9.358
ID-2 / IPS-88715	hand	femur	3 days	10.662	1.482	15.799
ID-2 / IPS-88715	hand	femur	30 days	9.930	1.283	12.741
ID-2 / IPS-88715	hand	femur	13 weeks	9.741	1.000	9.741
ID-2 / IPS-88715	hand	femur	15 weeks	2.006	0.960	1.926
ID-3 / IPS-93664	hand	femur	13 weeks	9.727	1.000	9.727
ID-3 / IPS-93664	hand	femur	15 weeks	4.570	0.971	4.435
ID-3 / IPS-93664	hand	femur	17 weeks	7.438	0.918	6.828
ID-3 / IPS-93664	hand	femur	23 weeks	4.125	0.794	3.274
ID-4 / IPS-88713	hand	femur	9 weeks	6.874	1.100	7.562
ID-4 / IPS-88713	hand	femur	11 weeks	11.132	1.021	11.361
ID-4 / IPS-88713	hand	femur	13 weeks	5.708	1.000	5.708
ID-4 / IPS-88713	hand	femur	15 weeks	3.425	0.985	3.374
ID-4 / IPS-88713	hand	femur	17 weeks	1.561	0.970	1.514
ID-4 / IPS-88713	hand	femur	23 weeks	4.978	0.879	4.377
ID-24 / IPS-109291	nursed	femur	13 weeks	8.683	1.000	8.683
ID-23 / IPS-109290	nursed	femur	13 weeks	3.035	1.000	3.035
ID-1 / IPS-88714	hand	tibia	Birth	12.951	1.114	14.429
ID-1 / IPS-88714	hand	tibia	3 days	17.396	1.087	18.907
ID-1 / IPS-88714	hand	tibia	15 days	4.152	1.021	4.237

ID-2 / IPS-88715	hand	tibia	3 days	12.290	0.999	12.277
ID-2 / IPS-88715	hand	tibia	30 days	6.764	0.913	6.176
ID-2 / IPS-88715	hand	tibia	13 weeks	7.059	1.000	7.059
ID-2 / IPS-88715	hand	tibia	15 weeks	3.240	0.661	2.143
ID-3 / IPS-93664	hand	tibia	13 weeks	7.701	1.000	7.701
ID-3 / IPS-93664	hand	tibia	15 weeks	7.886	0.678	5.347
ID-3 / IPS-93664	hand	tibia	17 weeks	4.107	0.653	2.681
ID-3 / IPS-93664	hand	tibia	23 weeks	9.200	0.555	5.103
ID-4 / IPS-88713	hand	tibia	9 weeks	5.340	0.767	4.097
ID-4 / IPS-88713	hand	tibia	11 weeks	16.607	0.724	12.021
ID-4 / IPS-88713	hand	tibia	13 weeks	4.251	1.000	4.251
ID-4 / IPS-88713	hand	tibia	15 weeks	2.699	0.692	1.867
ID-4 / IPS-88713	hand	tibia	17 weeks	0.628	0.668	0.420
ID-4 / IPS-88713	hand	tibia	23 weeks	6.177	0.605	3.739
ID-24 / IPS-109291	nursed	tibia	13 weeks	12.8987	1.000	12.899
ID-23 / IPS-109290	nursed	tibia	13 weeks	4.046	1.000	4.046
ID-1 / IPS-88714	hand	humerus	Birth	10.692	1.407	15.044
ID-1 / IPS-88714	hand	humerus	3 days	22.773	1.370	31.190
ID-1 / IPS-88714	hand	humerus	15 days	NA	1.354	NA
ID-2 / IPS-88715	hand	humerus	3 days	14.617	1.356	19.821
ID-2 / IPS-88715	hand	humerus	30 days	11.895	1.216	14.469
ID-2 / IPS-88715	hand	humerus	13 weeks	8.784	1.000	8.784
ID-2 / IPS-88715	hand	humerus	15 weeks	2.665	0.909	2.423
ID-3 / IPS-93664	hand	humerus	13 weeks	11.424	1.000	11.424
ID-3 / IPS-93664	hand	humerus	15 weeks	5.904	0.896	5.292
ID-3 / IPS-93664	hand	humerus	17 weeks	8.006	0.849	6.801
ID-3 / IPS-93664	hand	humerus	23 weeks	4.009	0.745	2.985

ID-4 / IPS-88713	hand	humerus	9 weeks	7.975	0.889	7.092
ID-4 / IPS-88713	hand	humerus	11 weeks	8.466	0.855	7.241
ID-4 / IPS-88713	hand	humerus	13 weeks	5.253	1.000	5.253
ID-4 / IPS-88713	hand	humerus	15 weeks	1.791	0.817	1.464
ID-4 / IPS-88713	hand	humerus	17 weeks	1.642	0.800	1.314
ID-4 / IPS-88713	hand	humerus	23 weeks	6.114	0.722	4.411
ID-24 / IPS-109291	nursed	humerus	13 weeks	10.377	1.000	10.377
ID-23 / IPS-109290	nursed	humerus	13 weeks	5.624	1.000	5.624
ID-1 / IPS-88714	hand	radius	Birth	10.223	1.721	17.592
ID-1 / IPS-88714	hand	radius	3 days	13.908	1.666	23.175
ID-1 / IPS-88714	hand	radius	15 days	NA	1.563	NA
ID-2 / IPS-88715	hand	radius	3 days	8.156	1.537	12.537
ID-2 / IPS-88715	hand	radius	30 days	5.985	1.407	8.423
ID-2 / IPS-88715	hand	radius	13 weeks	3.158	1.000	3.158
ID-2 / IPS-88715	hand	radius	15 weeks	4.202	1.063	4.468
ID-3 / IPS-93664	hand	radius	13 weeks	5.996	1.000	5.996
ID-3 / IPS-93664	hand	radius	15 weeks	4.503	0.971	4.372
ID-3 / IPS-93664	hand	radius	17 weeks	4.075	0.931	3.794
ID-3 / IPS-93664	hand	radius	23 weeks	4.503	0.838	3.774
ID-4 / IPS-88713	hand	radius	9 weeks	4.580	1.118	5.121
ID-4 / IPS-88713	hand	radius	11 weeks	7.772	1.079	8.389
ID-4 / IPS-88713	hand	radius	13 weeks	1.581	1.000	1.581
ID-4 / IPS-88713	hand	radius	15 weeks	0.684	1.053	0.720
ID-4 / IPS-88713	hand	radius	17 weeks	0.411	1.036	0.426
ID-4 / IPS-88713	hand	radius	23 weeks	3.492	0.952	3.325
ID-24 / IPS-109291	nursed	radius	13 weeks	2.922	1.000	2.922
ID-23 / IPS-109290	nursed	radius	13 weeks	1.999	1.000	1.999

ID-1 / IPS-88714	hand	metatarsus	Birth	13.307	1.289	17.156
ID-1 / IPS-88714	hand	metatarsus	3 days	19.602	1.256	24.629
ID-1 / IPS-88714	hand	metatarsus	15 days	1.622	1.176	1.907
ID-2 / IPS-88715	hand	metatarsus	3 days	9.129	1.264	11.537
ID-2 / IPS-88715	hand	metatarsus	30 days	4.484	1.177	5.278
ID-2 / IPS-88715	hand	metatarsus	13 weeks	6.439	1.000	6.439
ID-2 / IPS-88715	hand	metatarsus	15 weeks	5.337	0.820	4.375
ID-3 / IPS-93664	hand	metatarsus	13 weeks	NA	1.000	NA
ID-3 / IPS-93664	hand	metatarsus	15 weeks	12.791	0.842	10.768
ID-3 / IPS-93664	hand	metatarsus	17 weeks	NA	0.811	NA
ID-3 / IPS-93664	hand	metatarsus	23 weeks	12.008	0.670	8.047
ID-4 / IPS-88713	hand	metatarsus	9 weeks	5.144	1.091	5.614
ID-4 / IPS-88713	hand	metatarsus	11 weeks	NA	1.041	NA
ID-4 / IPS-88713	hand	metatarsus	13 weeks	NA	1.000	NA
ID-4 / IPS-88713	hand	metatarsus	15 weeks	NA	0.992	NA
ID-4 / IPS-88713	hand	metatarsus	17 weeks	NA	0.948	NA
ID-4 / IPS-88713	hand	metatarsus	23 weeks	6.434	0.822	5.287
ID-24 / IPS-109291	nursed	metatarsus	13 weeks	6.31	1.000	6.310
ID-23 / IPS-109290	nursed	metatarsus	13 weeks	5.310	1.000	5.310
ID-1 / IPS-88714	hand	metacarpus	Birth	12.702	1.972	25.052
ID-1 / IPS-88714	hand	metacarpus	3 days	17.669	1.924	34.004
ID-1 / IPS-88714	hand	metacarpus	15 days	NA	1.826	NA
ID-2 / IPS-88715	hand	metacarpus	3 days	8.848	1.969	17.419
ID-2 / IPS-88715	hand	metacarpus	30 days	1.745	1.874	3.270
ID-2 / IPS-88715	hand	metacarpus	13 weeks	2.516	1.000	2.516
ID-2 / IPS-88715	hand	metacarpus	15 weeks	2.772	1.389	3.851
ID-3 / IPS-93664	hand	metacarpus	13 weeks	NA	1.000	NA

ID-3 / IPS-93664	hand	metacarpus	15 weeks	10.977	1.385	15.207
ID-3 / IPS-93664	hand	metacarpus	17 weeks	NA	1.360	NA
ID-3 / IPS-93664	hand	metacarpus	23 weeks	6.176	1.203	7.432
ID-4 / IPS-88713	hand	metacarpus	9 weeks	4.367	1.845	8.056
ID-4 / IPS-88713	hand	metacarpus	11 weeks	NA	1.789	NA
ID-4 / IPS-88713	hand	metacarpus	13 weeks	NA	1.000	NA
ID-4 / IPS-88713	hand	metacarpus	15 weeks	NA	1.702	NA
ID-4 / IPS-88713	hand	metacarpus	17 weeks	NA	1.659	NA
ID-4 / IPS-88713	hand	metacarpus	23 weeks	6.474	1.482	9.592
ID-24 / IPS-109291	nursed	metacarpus	13 weeks	7.571	1.000	7.571
ID-23 / IPS-109290	nursed	metacarpus	13 weeks	5.290	1.000	5.290
